# A tannin compound from *Sanguisorba officinalis* blocks Wnt/β-catenin signaling pathway and induces apoptosis of colorectal cancer cells

**DOI:** 10.1186/s13020-019-0244-y

**Published:** 2019-05-31

**Authors:** Wa Li, Chun-juan Yang, Li-qian Wang, Juan Wu, Cong Dai, Yue-mei Yuan, George Q. Li, Mei-cun Yao

**Affiliations:** 1 School of Pharmaceutical Sciences, Sun Yat-sen University, Guangzhou, 510006 People’s Republic of China; 2 Department of Pharmaceutical Analysis and Analytical Chemistry, College of Pharmacy, Harbin Medical University, Harbin, 150081 Heilongjiang China; 3 School of Pharmacy, Shenyang Pharmaceutical University, Shenyang, 110016, China; 4 NICM Health Research Institute, Western Sydney University, Locked Bag 1797, Penrith, NSW 2751 Australia

**Keywords:** *Sanguisorba officinalis*, Wnt/β-catenin, Colorectal cancer, Apoptosis, Transcriptomics, 1,4,6-Tri-*O*-galloyl-β-d-glucopyranose

## Abstract

**Background:**

*Sanguisorba officinalis*, a popular Chinese herb, called DiYu, has been shown to inhibit the growth of many human cancer cell lines, including colorectal cancer cells. The aims of this study were to discover the active compound and molecular mechanism of *S. officinalis* against Wnt/β-catenin signaling pathway and develop Wnt inhibitors from natural products as anti-colorectal cancer agents.

**Methods:**

1,4,6-Tri-*O*-galloyl-β-d-glucopyranose (TGG) was obtained by the preparative HPLC. The effect of DiYu on proliferation of NIH3T3 and HT29 was detected by MTT assay. Luciferase reporter assay was applied to investigate the activity of Wnt/β-catenin signaling in NIH3T3. The expression levels of mRNA and protein were detected by RT-PCR and western blot. Immunofluorescence assay was used to measure the level of β-catenin in cytoplasm and nucleus. Transcriptomic profiling study was performed to investigate the molecular mechanism of DiYu on the Wnt/β-catenin signaling pathway.

**Results:**

TGG significantly inhibited the Wnt/β-catenin signaling pathway, down-regulated the expression of β-catenin and Wnt target genes (Dkk1, c-Myc, FGF20, NKD1, Survivin), up-regulated the levels of cleaved caspase3, cleaved PARP and ratio of Bax/Bcl-2, which may explain the apoptosis of HT29.

**Conclusions:**

Our study enhanced the discovery of the materials and elucidation of mechanisms that account for the anti-Wnt activity of natural inhibitor (DiYu) and identified the potential of TGG to be developed as anti-colorectal cancer drugs.

**Electronic supplementary material:**

The online version of this article (10.1186/s13020-019-0244-y) contains supplementary material, which is available to authorized users.

## Background

Colorectal cancer (CRC) is the third most commonly diagnosed cancer in the world, which is closely related to the aberrant activation of Wnt/β-catenin signaling pathway [1–3]. It was found that most mutations of Wnt/β-catenin signaling were caused by the dysfunction of Apc [4] or β-catenin [5]. They are respectively the negative and effector proteins involved in the Wnt signaling. A large number of potent chemotherapeutic agents in clinical practice on CRC display a narrow therapeutic window and drug resistance. 5-FU and CPT-11 are the first-line anti-colorectal cancer drugs widely used in clinic, but with the emergence of drug resistance and toxic side effects, their clinical efficacy is unsatisfactory [6, 7]. Currently, CRC remains a major cause of disease and death in most countries. Thus, there is an urgent need to develop effective and safe agents for the treatment of CRC. One alternative approach is to develop Wnt inhibitors from natural plants for anti-CRC drug development.

Wnt/β-catenin signaling, as a proliferative and self-renewal signaling pathway, is often complicated in the stem cell control [8]. Excessive activation of the Wnt/β-catenin signaling leads to specific human diseases [9], including CRC [1]. In the absence of Wnt protein, the degradation of β-catenin is regulated by a triplet, which is composed of Axin protein, adenomatous polyposis coli (Apc) and glycogen synthase kinase 3 (Gsk3β) [10]. The Gsk3β sequentially phosphorylates the amino terminal region of β-catenin, contributing to β-catenin ubiquitination and proteasomal degradation, resulting in the elimination of β-catenin and the suppression of Wnt target genes. Moreover, the Wnt/β-catenin signaling pathway could be activated under the Wnt ligand binding to the seven-pass transmembrane Frizzled (Fz) receptor [11] and its co-receptor, a low-density lipoprotein receptor related protein 5/6(LRP5/6) [12]. The formation of Wnt-Fz-LRP6 complex together with the scaffolding protein Dishevelled (Dvl) leads to inhibition of β-catenin phosphorylation. These events result in the stabilization and accumulation of β-catenin, which travels to the nucleus engaging T cell factor/lymphoid enhancer factor (TCF/LEF) [13] to activate Wnt target gene expression.

*Sanguisorba officinalis* (also called great burnet) is a common Chinese herbal medicine from the family Rosaceae, used to treat hemostasis and inflammation. It has broad biological activities including anti-cancer, anti-inflammatory, and anti-oxidative activities [14]. Our previous results have proved that the aqueous extract of great burnet showed strong inhibition against proliferation of CRC cells (HCT116, RKO) [15] and Wnt/β-catenin signaling pathway [16]. However, the active compound and the underlying molecular mechanism such as apoptosis process in HT29 cells remained unknown.

Therefore, the aims of this study were to discover the active compound and molecular mechanism of *S. officinalis* against Wnt/β-catenin signaling pathway, and develop Wnt inhibitors from natural products as anti-colorectal cancer agents.

## Materials and methods

Additional file 1: Minimum Standards of Reporting Checklist contains details of the experimental design, and statistics, and resources used in this study.

### Plant materials

The *Sanguisorba officinalis* root pieces studied in this research were purchased from Zisun Pharmaceutical Companies (Guangzhou, China) (voucher numbers: 20160101) which were authenticated by Prof. Depo Yang (Sun Yat-sen University) and the corresponding voucher specimens were stored in the Pharmaceutical Analysis and Quality Assessment Laboratory, School of Pharmaceutical Sciences, Sun Yat-sen University, China.

### Extraction and isolation of 1,4,6-Tri-*O*-galloyl-β-d-glucopyranose (TGG)

Dried rhizome of *S. officinalis* was boiled and refluxed with ultrapure water for 1 h (three times). After filtration, the extracted solution was evaporated under reduced pressure and the condensate was subjected to D101 macroporous resin column chromatography, eluted with water, 40% ethanol, 95% ethanol to afford DY-A, DY-B, DY-C. DY-B was further purified through preparative HPLC eluting with 40% aqueous acetonitrile to afford the compound DYB4, which was identified as 1,4,6-tri-*O*-galloyl-β-d-glucopyranose (TGG) by 1H-NMR and 13C-NMR. 1H-NMR assigns the signals to the different protons of the molecule, 13C-NMR assigns the signals to the different carbon atoms of the molecule.

### Chemicals and reagents

The powder of 3-(4,5-dimethylthiazol-2-yl)-2,5-diphenyltetrazolium bromide was purchased from MP Biomedicals (Santa Ana, USA). DEPC solution was obtained from Sangon Biotech (Shanghai, China). Wnt3a protein was obtained from Stem RD (Burlingame, USA). Steady-Glo^®^ luciferase assay system kit was from Promega Corporation (Madison, USA). PrimeSCript™RT reagent kit with gDNA Eraser and SYBR^®^ Premix Ex Taq™ II (Tli RNaseH Plus) were purchased from Takara Bio Inc (Kusatsu, Shiga, Japan). The anti-β-catenin (#19807), anti-β-actin (#4970), anti-Bax (#14796), anti-Bcl-2 (#4223), anti-caspase3 (#9662), anti-PARP (#9532) and RIPA reagent were purchased from Cell Signaling Technology, Inc. (Danvers, MA, USA). The second antibody (#AP132P) was from EMD Millipore Corporation (Temecula, CA). Alexa Fluor 594 AFFINIpure Goat Anti-Rabbit IgG(H+L) and DAPI Fluoromount-Gtm were purchased from Yeasen Co., Ltd (Shanghai, China).

### Cell lines and culture

The NIH3T3 cells transfected with stable top flash plasmid were purchased from Curegenix Inc (Guangzhou, China). HT29 cells were a gift from Professor Jun Du (Sun Yat-sen University, China). Both cell lines were cultured in DMEM which contains 10% fetal bovine serum (Gibco, China) in the incubator of 37 °C and 5% CO_2_.

### Cell viability

NIH3T3 and HT29 cells were plated in 96-well plates at a concentration of 8000 cells/well for 24 h. Then cells were treated with culture medium supplemented with different drug solution. After 24 h co-cultivation, 3-(4,5-dimethylthiazol-2-yl)-2,5-diphenyltetrazolium bromide (MTT) was added to evaluate the viability of cells.

### Luciferase reporter assays

NIH3T3 cells were plated in 96-well plates at a concentration of 2 × 10^4^ cells/well. After 24 h, cells were treated with DMEM medium containing different *S. officinalis* drug solutions in the presence of wnt3a (100 ng/mL) for another 24 h. After the treatment, cell luciferase activity was detected according to the manufacturer’s instruction of Steady-Glo^®^ luciferase assay system kit.

### Western blotting assay

NIH3T3 and HT29 cells were seeded in 6-well plates with 3 × 10^5^ cells/well overnight, treated with different drug solution in DMEM medium. After 24 h incubation, cells were digested by trypsin and centrifuged 5 min at 1000 rpm, the deposit was lysed with RIPA reagent on ice to obtain the total cellular protein. The concentration of proteins was determined using bicinchoninic acid assay (BCA). The targeted protein bands were visualized by SuperSignal™ west Pico Chemiluminescent Substrate (Thermo Fisher Scientific, MA, USA) and imaged by the ChemiDoc XRS+ system (Bio-Rad, Hercules, CA, USA). The expression profile of different tested proteins was analyzed under the same experimental conditions. And a single western blot experiment in each case was performed because that some tested proteins have similar molecular weight.

### Reverse transcription quantitative PCR assay

Both two cell lines were plated in 6-well plates at a density of 3 × 10^5^ cells/well to measure the expression level of Wnt target gene or its related gene by quantitative real time PCR assay. After incubated with different drug solution for 10 h, cells were lysed by Trizol reagent (Invitrogen, USA) to extract the total RNA. The RNA concentration of each sample was determined with the Qubit2.0 at 260/280 nm (Thermo Fisher Scientific, Waltham, MA, USA), then the purified total RNA was used to perform the reverse transcription to obtain cDNA according to the manufacture’s protocol of PrimeScript RT reagent Kit (Takara). The cDNA was mixed with other reaction reagent of SYBR Premix Ex Tap™ kit (Takara) and PCR Forward/Reverse Primer to perform the quantitative PCR assay on PCR system (Bio-Rad, CFX96, USA), the obtained data were analyzed using ΔΔCt approach. The several genes primers sequences were showed in Additional file 2: Table S1.

### Immunofluorescence assay

NIH3T3 and HT29 cells were plated in 15 mm confocal dishes for 8 × 10^4^ cells/well overnight, which were then treated with the drug solution for 24 h. Each sample was washed with PBS and fixed by 4% paraformaldehyde for 30 min. Immediately, the fixative liquid was removed by PBS and the 0.1% Triton X-100 was added to permeabilize cells. Subsequently, cells were blocked with 5% skim milk solution and blotted using the anti-β-catenin in the same way as western blot assay, following the incubation of cells with the IgG labeled anti-fluorescence. Finally, the cell nucleus was stained with DAPI Fluoromount-G™, which was observed and imaged under the confocal microscopy (Zeiss, Smartproof 5, Germany).

### Transcriptomics analysis

For the sequencing analysis, NIH3T3 cells seeded in 6-well plate were treated with drug solution for 10 h and lysed with Trizol reagent. The cell transcriptomics was analyzed by the Biomarker Technologies (Beijing, China). The processing protocol was as follows: first, RNA degradation and contamination were monitored by using 1% agarose gels, the purity of which was determined with NanoPhotometer spectrophotometer (IMPLEN, CA, USA). Then the RNA concentration was measured according to the instruction of Qubit RNA Assay Kit (Life Technologies, CA, USA), at the same time, the integrity of RNA was measured by RNA Nano 6000 Assay Kit (Agilent Technologies, CA, USA). Each RNA sample including 1 μg RNA was prepared to establish the sequencing libraries by NEBNext UltraTM RNA Library Prep Kit. Finally, according to the established library, the sequencing was conducted by using the way of illumine Hiseq 2500 platform and generating the paired-end reads. It was needed for raw data to remove the reads containing adapter and low quality before analysis. Additionally, the differential expression analysis of genes between any two samples was performed by the DEGseq (2010) R package. Q value < 0.005 and |log2 (fold change)| ≥ 1 was considered to be significantly differential expression. KEGG pathway analysis and GO enrichment analysis of DEGs were performed by the KOBAS 3.0 software and GOseq R packages (http://www.geneontology.org/).

### Flow cytometry analysis of apoptosis and the cell cycle

For the analysis of apoptosis, cells were washed by cold PBS, then prepared according to the Annexin V-FITC Apoptosis Detection Kit protocol. In cell cycle assays, cells were washed by cold PBS after the treatment with drugs. Then pre-cooled 70% ethanol (stored at − 20 °C overnight) was added to fix cells. Finally, cells were treated with PI/RNase solution for 15 min after washing with PBS. And the Coulter Epics XL Flow Cytometric System (Beckman Coulter, Miami, FL, USA) was used to detect the samples.

### Statistics analysis

All experiments except the transcriptomics analysis in this study were repeated in triplicates and their data were expressed as mean ± SD (*n *= 3). The statistics analysis was conducted by Graph prism 5 software. The significant difference was evaluated by two-tailed Student’s *t* test and *P* < 0.05 was a representative of statistically significant difference.

## Results

### *Sanguisorba officinalis* and the active component blocked Wnt/β-catenin signaling pathway in NIH3T3 cells

**Fig. 1:**
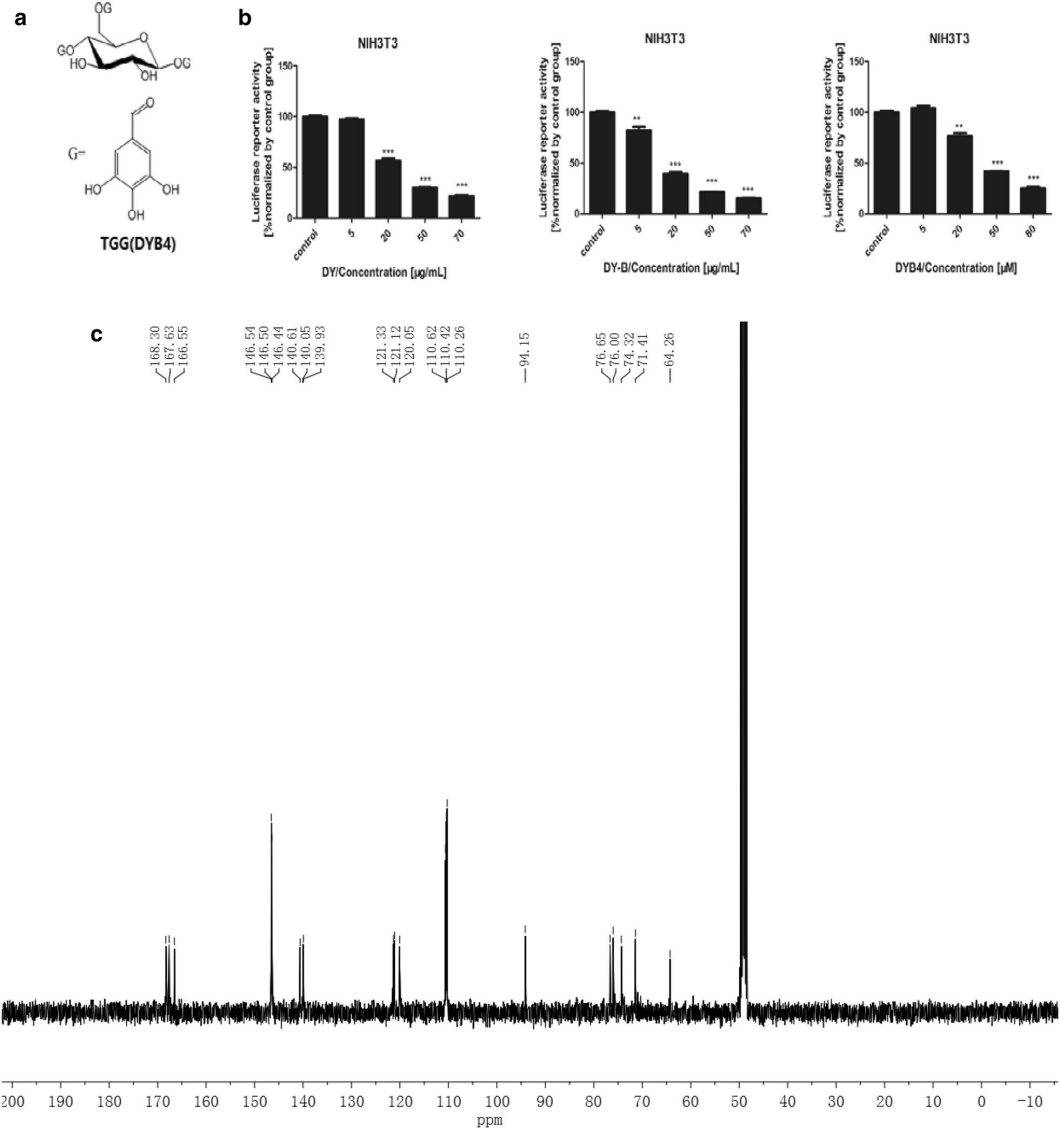
Inhibitory effects of DY, DY-B, DYB4 on the Wnt/β-catenin signaling pathway in NIH3T3 cells. **a** The chemical structure of DYB4. **b** The dose-dependently inhibition by DY, DY-B, DYB4 on the Wnt/β-catenin signaling pathway. **c** The 13C-NMR information of DYB4. **P < 0.01, ***P < 0.001, vs. control group

**Fig. 2:**
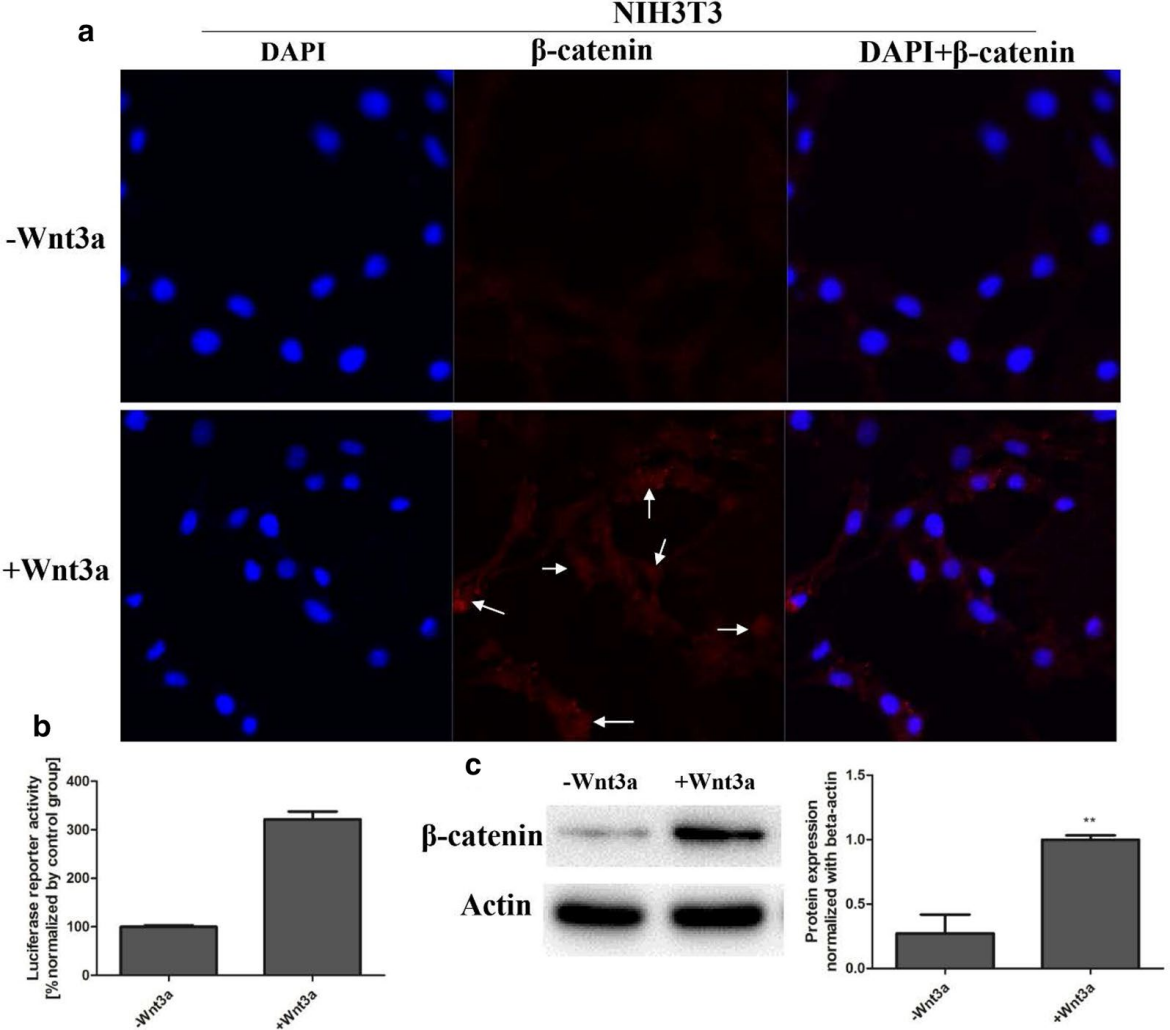
The effect on the Wnt signaling pathway in NIH3T3 cells treated with Wnt3a. **a** The influences on β-catenin in different parts of NIH3T3 cells by Immunofluorescence assays. The level of β-catenin in nucleus was up-regulated which was indicated by the arrow. **b** The luciferase reporter assay showed that the Wnt signal was activated in NIH3T3 cells treated with Wnt3a. **c** The expression level of β-catenin was up-regulated in NIH3T3 cells treated with Wnt3a by western blot

To identify the natural products from traditional medicine *Sanguisorba officinalis* that blocked the Wnt signaling pathway, wnt3a-treated NIH3T3 cells model is important for evaluating the drug effects on Wnt/β-catenin signaling pathway, which were transfected with top flash plasmid containing TCF/LEF regions [13] for the luciferase reporter assay. NIH3T3 cells were first incubated with the aqueous extract DY and ethanol elution of gained from a D101 macroporous absorbent resin column in the presence of Wnt3a conditioned medium, and the activity of drugs was determined by the MTT assay (Additional file 2: Figure S6), followed by luciferase reporter assay. The results showed that the aqueous extract DY and the 40% ethanol elution DY-B dose-dependently inhibited the Wnt signaling pathway (Fig. 1b). The Wnt signal was up-regulated in NIH3T3 cells treated with Wnt3a (Fig. 2). Obtained as the tan amorphous powder, DY-B was subsequently purified by a preparative HPLC for monomers, and activities of the monomers were also screened by the luciferase reporter assay (Additional file 2: Figure S1). Using this strategy, an active compound DYB4 of good inhibitory activity on the Wnt signaling pathway was isolated (Fig. 1b), and the purity of DYB4 was analyzed by HPLC. The structure was determined with ^13^C-NMR and ^1^H-NMR (Additional file 2: Figure S9) as 1,4,6-tri-*O*-galloyl-β-d-glucopyranose (TGG) [17] (Fig. 1a). 1H-NMR assigns the signals to the different protons of the molecule, 13C-NMR assigns the signals to the different carbon atoms of the molecule.

**Fig. 3:**
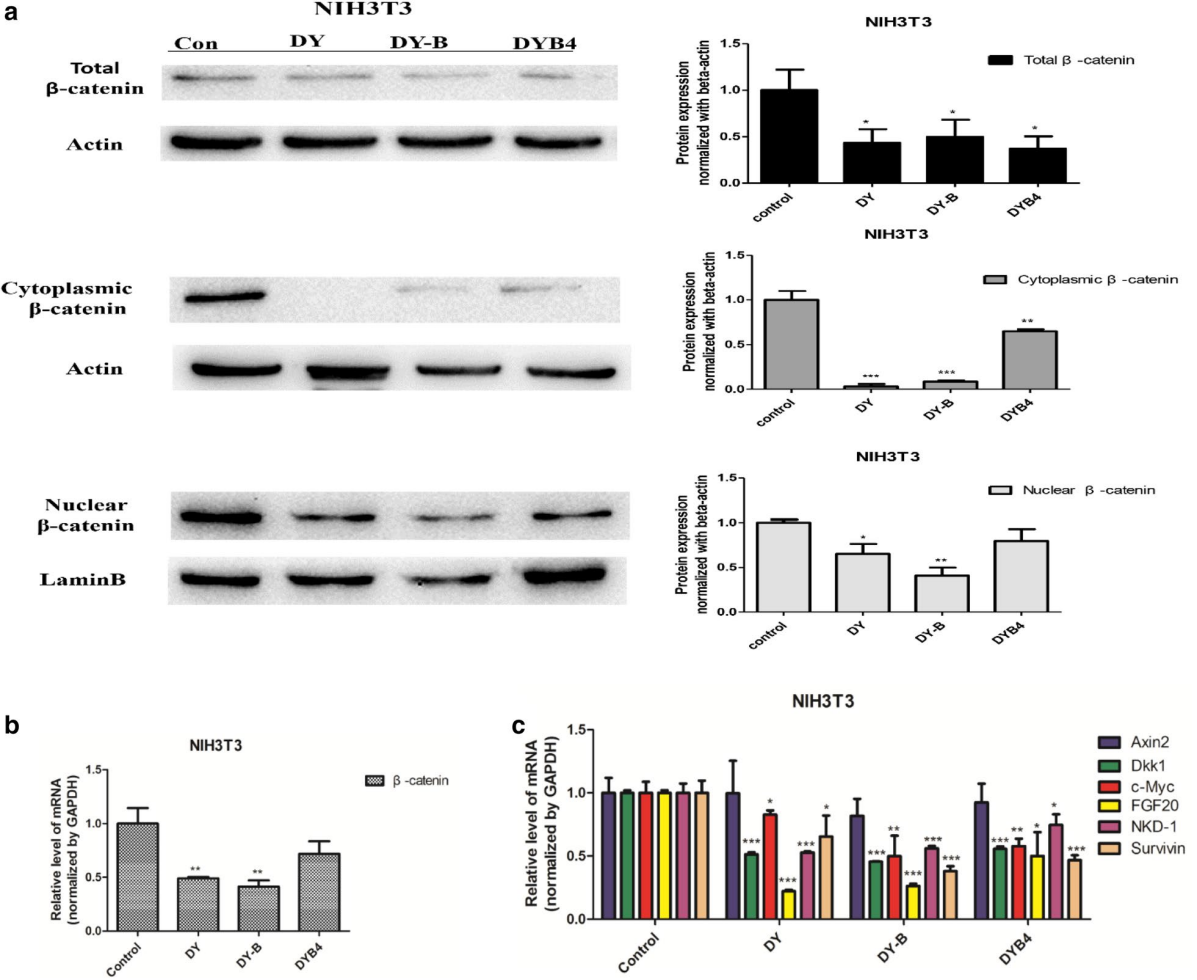
Effects of DY, DY-B, DYB4 on the β-catenin and Wnt target genes in NIH3T3 cells. **a**–**c** Respectively suggest that DY (40 μg/mL), DY-B (40 μg/mL), DYB4 (40 μM) down- regulated the expression of β-catenin and Wnt target genes (Dkk1, c-Myc, FGF20, NKD-1, Survivin). *P < 0.05, **P < 0.01, ***P < 0.001, vs. control group

In order to further confirm that the DY, DY-B and DYB4 have strong inhibitory effects on the Wnt/β-catenin signaling pathway, we studied the influence of these products on the expression of Wnt signaling key protein, β-catenin. The active β-catenin plays an important role in the Wnt/β-catenin signaling pathway, by entering the nucleus and binding to the TCF/LEF to activate the pathway when it is not phosphorylated and degraded by the complex [18]. Here, we studied the influence of DY, DY-B and DYB4 on β-catenin protein in different parts: the whole cell, cytoplasm and nucleus [19]. The western blot results showed that DY, DY-B and DYB4 significantly downregulated the level of β-catenin protein in these three parts (Fig. 3a).

Besides, to intuitively observe the effects on β-catenin protein by DY, DY-B and DYB4, the immunofluorescence assay was conducted, and the data indicated that the protein amounts were obviously decreased in the cytoplasm although it was not observed that β-catenin was successfully transferred into nucleus (Fig. 5a). Meanwhile, we performed the qPCR experiment to analyze the effect on mRNA level of β-catenin gene and other Wnt target gene. The results showed that the mRNA level of β-catenin was dramatically down-regulated by DY, DY-B and DYB4 (Fig. 3b), which revealed that these drugs may inhibit the Wnt signaling pathway by regulating the transcription and translation process of β-catenin gene. Furthermore, the mRNA levels of Wnt target genes (Dkk1 [20], c-Myc [21], FGF20, NKD-1 [22], Survivin [23]) were reduced, except for gene Axin2 [24], DY, DY-B and DYB4 didn’t show significant inhibitory effects on Axin2 gene (Fig. 3c).

### DY, DY-B and DYB4 down-regulated the Wnt/β-catenin signaling in HT29 cells

**Fig. 4:**
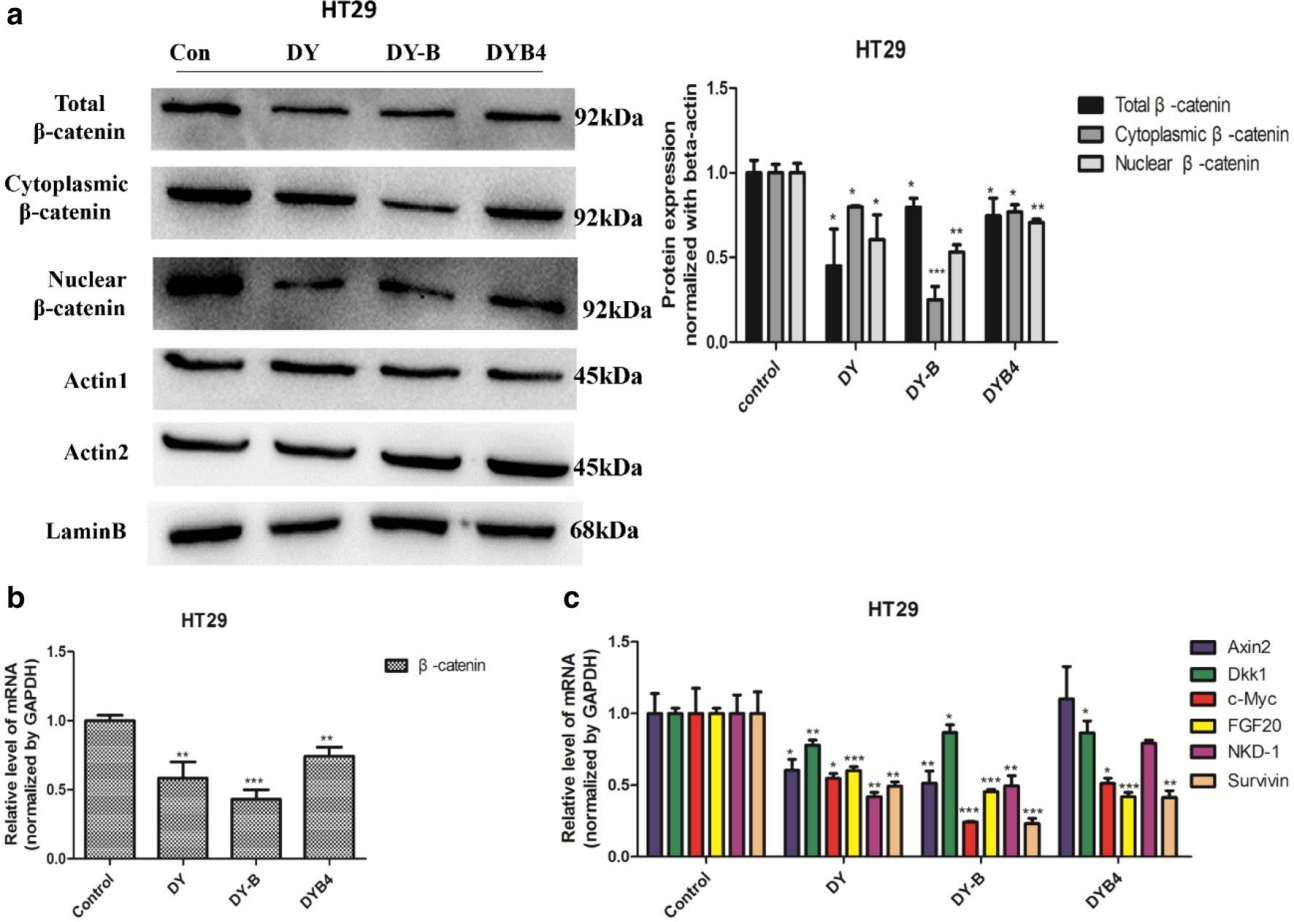
Effects of DY, DY-B, DYB4 on the β-catenin and Wnt target genes in colorectal cancer cell (HT29). **a**–**c** Respectively suggest that DY (40 μg/mL), DY-B (40 μg/mL) down- regulated the expression of β-catenin and Wnt target genes (Axin2, Dkk1, c-Myc, FGF20, NKD-1, Survivin), DYB4 (40 μM) also down-regulated the level of β-catenin and the mRNA of Dkk1, c-Myc, FGF20, Survivin. *P < 0.05, **P < 0.01, ***P < 0.001, vs. control group

**Fig. 5:**
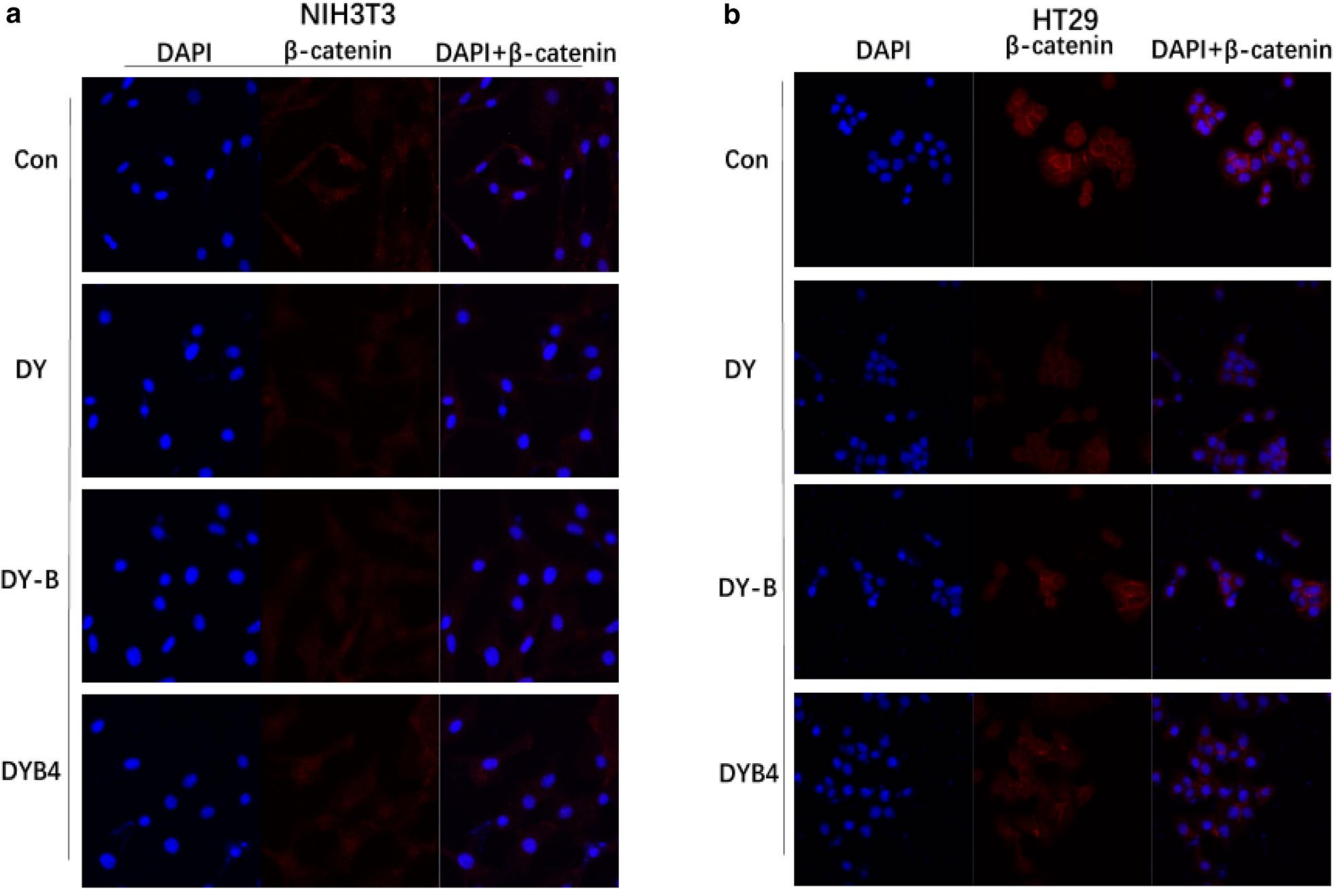
Effects of DY, DY-B and DYB4 on β-catenin levels in the cytoplasm and nucleus of NIH3T3 and HT29 cells by Immunofluorescence assays. **a**, **b** Showed the influence of DY (40 μg/mL), DY-B (40 μg/mL), DYB4 (40 μM) on the β-catenin in different parts of NIH3T3 and HT29 cells. The nucleus was stained with DAPI, and the subcellular localization of β-catenin (red) was detected by anti-β-catenin

Recent research manifested that the aberrant activation Wnt/β-catenin signaling pathway was closely related to colon cancer, and the mutation of Wnt signaling pathway genes (Apc, Axin2) were found in mostly intermittent colorectal cancer. Based on the previous data that DY, DY-B and DYB4 of *S. officinalis* showed a significant inhibitory activity on the Wnt signaling pathway in NIH3T3 cells, we further studied their influences on the colorectal cancer cell HT29. The concentrations of these drugs incubated with HT29 were determined by the MTT assay to avoid the impact of cells viabilities on the results (Additional file 2: Figure S7). The western blot assay results showed that DY, DY-B and DYB4 effectively down-regulated the levels of total β-catenin protein and the nucleus β-catenin protein, as well as the protein in the cytoplasm, in addition, the down-regulation effect of DYB4 on β-catenin protein was weaker than the DY and DY-B (Fig. 4a). The qPCR experiment results also validated the above results, the levels of β-catenin mRNA transcription were decreased by DY, DY-B and DYB4 (Fig. 4b). The immunofluorescence assay was also conducted in HT29, and the results demonstrated that the amounts of β-catenin protein were notably decreased by DY, DY-B and DYB4 (Fig. 5b) in cytoplasm, without affecting the nuclear translocation of β-catenin protein, which was similar to the results in NIH3T3 cells. For further confirmation, the Wnt target genes levels (Axin2 [24], Dkk1 [20], c-Myc [21], FGF20, NKD-1 [22], Survivin [23]) were also evaluated through the quantitative real time PCR assay in HT29 cells. These results demonstrated that DY and DY-B effectively reduced the transcription of the six target genes, and DYB4 only downregulated the expression of Dkk1, c-Myc, FGF20 and Survivin genes, but it had no effect on the expression of Axin2 and NKD-1 genes (Fig. 4c).

### The transcriptomics study of DY, DY-B and DYB4 in NIH3T3 cells

To further verify our findings and investigate the underlying molecular mechanism of DY, DY-B and DYB4 on the Wnt signaling pathway, the high-depth next generation sequencing study on NIH3T3 cells transfected with the top flash plasmid were performed, which was in the presence of Wnt3a ligand. The transcriptomics results showed that the gene expression profiles of NIH3T3 cells treated with, DY, DY-B and DYB4 were clearly different from the control group. We used EBseq to identify the statistically significant differential expression genes (DEGs) for pairwise comparisons between different cell groups. Results suggested that many differential expressions of genes were induced by these drugs. Different from the untreated cell group, 411 genes’ expressions were effectively altered in the cell group treated with DY, and 1012 and 1119 by DY-B and DYB4, respectively. Furthermore, we observed that the up-regulated DEGs was more than two times as many as the down-regulated DEGs (282 to 129) in cell group treated with DY. Interestingly, for all DEGs in the cell groups treated with DY-B and DYB4, approximately 80% (807 of 1012 and 936 of 1119) of these genes were identified as up-regulated DEGs, with few (205 and 183) being down-regulated DEGs as well (Table 1). The analysis of MA plot and Volcano plot was performed to show the significance of DEGs (Additional file 2: Figure S3). These results showed the DEGs induced by DY, DY-B, DYB4 may be related to the inhibition of Wnt signaling pathway.

**Table 1:** The numbers of DEGs induced by DY, DY-B and DYB4

DEG set	DEG number	Up-regulated	Down-regulated
Control-vs-DY	411	282	129
Control-vs-DY-B	1012	807	205
Control-vs-DYB4	1119	936	183

Based on the DEGs’ functional similarity, we analyzed the multiple biological functions of DY, DY-B and DYB4, including biological process, molecular function and cellular component. At the background of all genes, the secondary functions enrichment results of DEGs were showed in the Additional file 2: Figure S10. Comparing the top 10 secondary functions of biological process in different drugs groups, we found that the enrichment of intracellular signal transduction [25] was induced by DY and DY-B, which was not observed in DYB4 group. The GO term of the positive regulation of transcription [26] by DNA-templated enrichment was only found in DY and DY-B groups, in addition, the positive regulation of cell proliferation enrichment was only noticed in DY-B and DYB4 groups. In terms of molecular function and cellular component, the levels of DEGs enrichment exhibited difference in different groups. The heparin binding [27] and dendrite enrichments [28] were observed in DY group, the ubiquitin-protein transferase activity and lamellipodium enrichments were noticed in DY-B group, besides, the protein serine/threonine kinase activity and melanosome enrichments were found in DYB4 group (Additional file 2: Figure S4). The differentially expressed biological functions can be regulated by Wnt signaling through different mechanisms.

In order to further explore the potential functional pathway of DEGs altered by DY, DY-B and DYB4, the Kyoto Encyclopedia of Genes and Genomes (KEGG) analysis for the all DEGs in cell groups were conducted. The number of DEGs that contributed to multiple biological pathways including Cellular Processes, Environment Information Processing, Genetic Information Processing, Human Diseases, Metabolism and Organismal Systems, were shown in Additional file 2: Figure S11. It was interesting to find that the metabolism enrichment contributed by DEGs, such as glycolysis/gluconeogenesis, carton metabolism and glycerophospholipid metabolism, was only found in the DY group. And the DEGs enrichment of Genetic Information Processing such as RNA degradation, ubiquitin mediated proteolysis, and Fanconi anemia pathway was only noticed in DY-B and DYB4 groups. From the statistic of pathway enrichment, we found that most DEGs were enriched in the pathways about cancer in all groups. In DY group, the PI3K-Akt signaling pathway contributed by more DEGs was shown as significantly over-represented (Additional file 2: Figure S11).

### The analysis of DEGs about Wnt/β-catenin signaling pathway

**Fig. 6:**
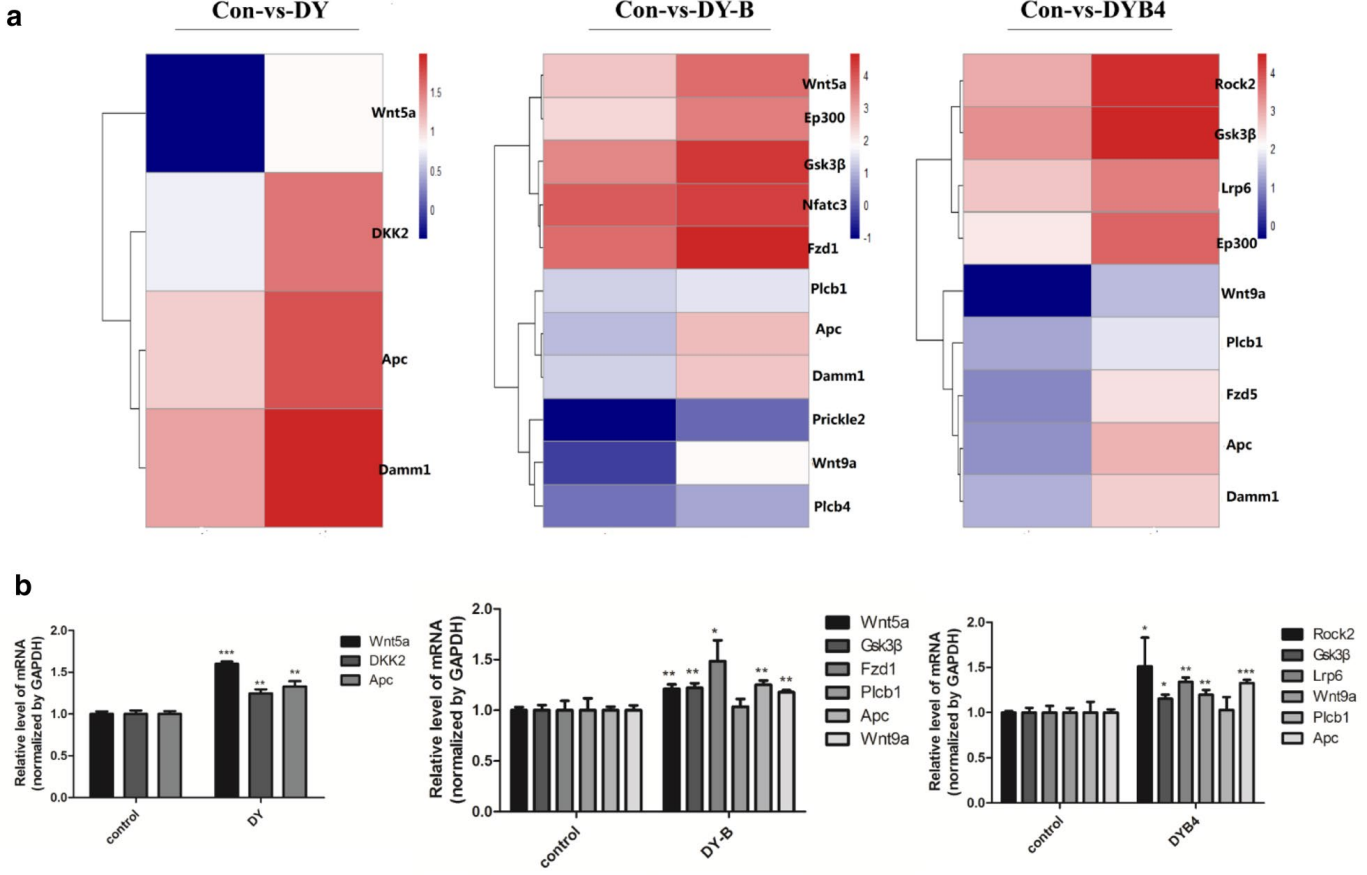
Effects of DY, DY-B, DYB4 on Wnt pathway-related genes. **a** The heatmaps of DEGs induced by DY, DY-B, DYB4, which were related to the Wnt/β-catenin signaling pathway of NIH3T3 cells. The down-regulated and up-regulated genes are presented in blue and red boxes, respectively; **b** The RT-PCR results of some genes of DY, DY-B, DYB4 groups to confirm the transcriptomics analysis

According to the KEGG analysis of DEGs in the Wnt/β-catenin signaling pathway, we compared the number of DEGs in the DY, DY-B and DYB4 groups, and it was interesting to find that all of DEGs were up-regulated. In DY group, only Wnt9a [29], Apc [10], Dkk2 [30] and Damm1 were observed as the up-regulated genes, while DY-B up-regulated the expression of Wnt9a, Apc, Damm1, Wnt5a, Gsk3β [10], Prickle2, Nfatc3, Plcb1, Plcb4, Fzd1, Ep300. And DYB4 up-regulated the expression of Wnt9a, Apc, Damm1, Gsk3β, Plcb1, Ep300, Rock2, Lrp6, Fzd5 (Fig. 6).

### The inhibition of Wnt/β-catenin signaling pathway by DY, DY-B, DYB4 contributed to apoptosis in HT29 cells

**Fig. 7:**
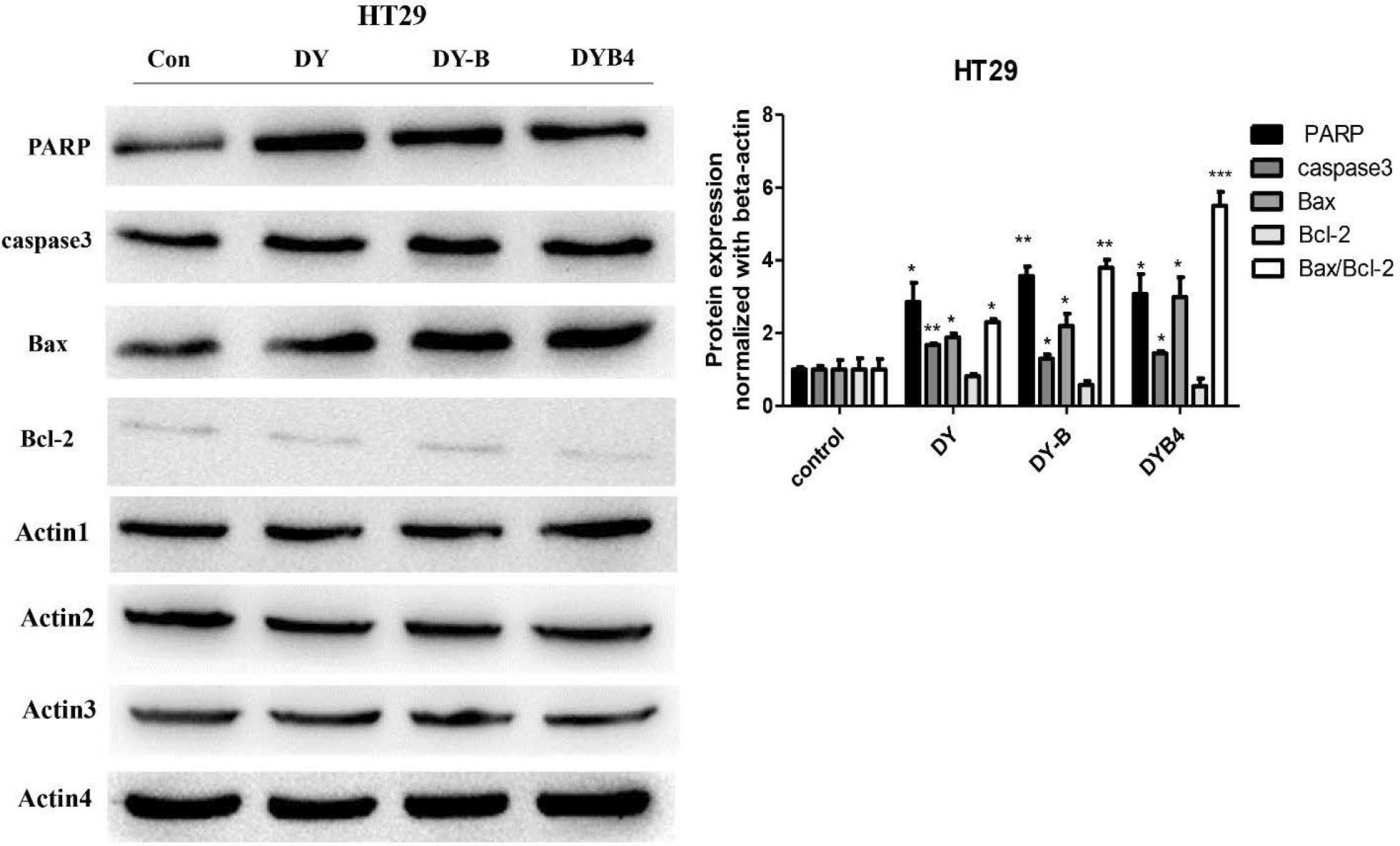
Effects of DY, DY-B, DYB4 on the apoptosis related genes. The figure indicates that DY (40 μg/mL), DY-B (40 μg/mL), DYB4 (40 μM) effectively up-regulated the level of caspase3, PARP, Bax, while had no obvious effect on Bcl-2. *P < 0.05, **P < 0.01, ***P < 0.001, vs. control group

**Fig. 8:**
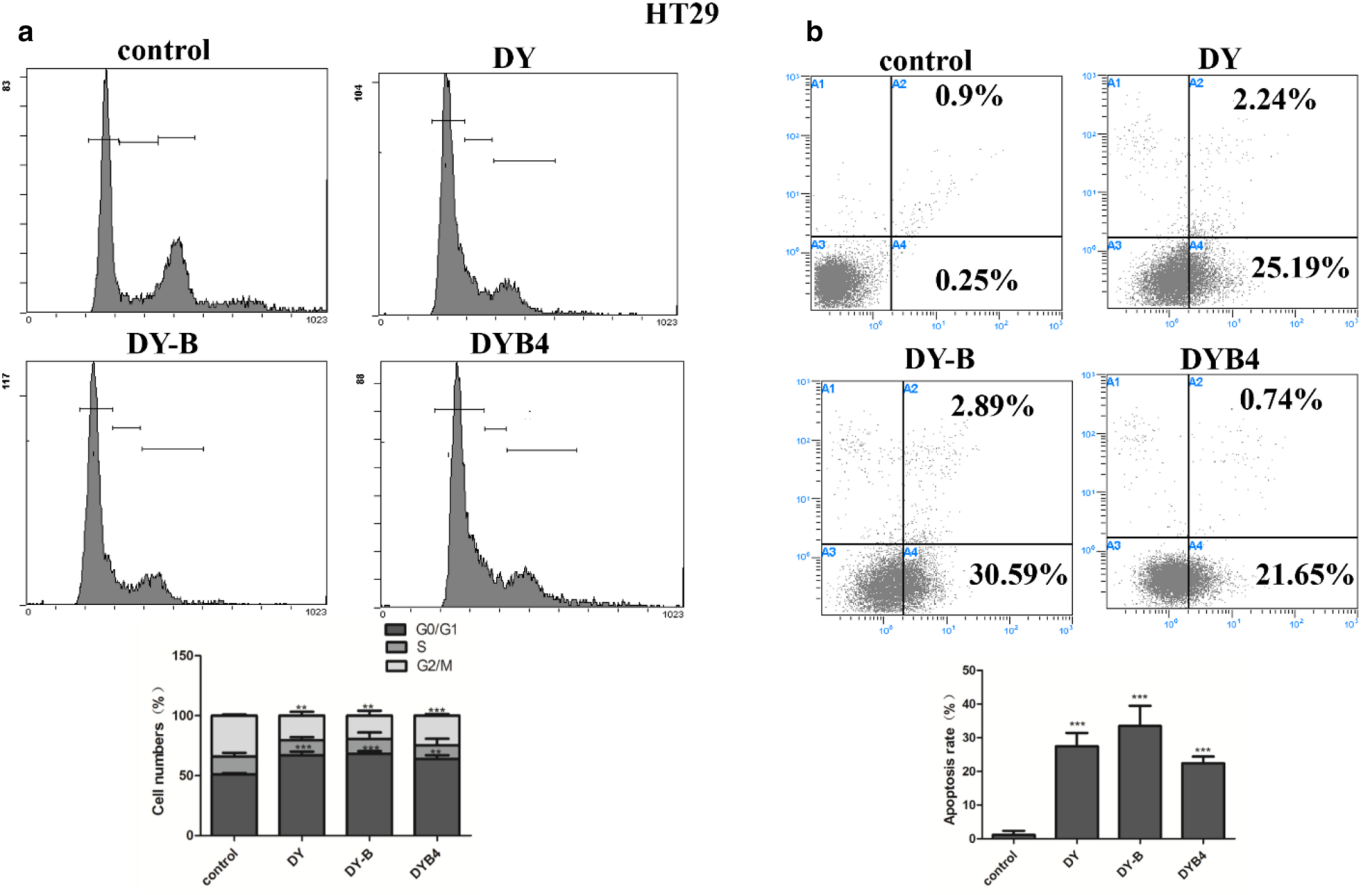
DY, DY-B, DYB4 induced apoptosis in HT29 cells. **a** Cells were stained by PI & RNase and DNA contents were then measured by flow cytometry system (FCMS). **b** Apoptotic CRC cells stained with Annexin V-FITC/PI were detected by FCMS, and apoptotic rate was showed below. All data are expressed as mean ± SD (n = 3), *P  < 0.05, **P  < 0.01, ***P  <  0.001, vs control group

Deregulation of Wnt/β-catenin signaling pathway was reported to be associated with cancer development and regulates the cell proliferation, differentiation and apoptosis [31, 32]. Based on our previous studies that DY can inhibit the proliferation of HCT116 and RKO [15], we investigated whether the inhibition of Wnt/β-catenin signaling pathway on HT29 treated with DY, DY-B, DYB4 could affect the cells apoptosis. Several key proteins’ expression (caspase3, PARP, Bax, Bcl-2) [31] in the process of apoptosis were studied by western blot assay. Data showed that the expression of caspase3, PARP and Bax were up-regulated, and although the down-regulation of Bcl-2 was not obvious in all groups, the level of Bax/Bcl-2 ratio was also increased (Fig. 7). Cell cycle experiments results showed that HT29 cells can be blocked in G0/G1 phase (Fig. 8a), and the apoptotic rate was obviously increased in HT29 cells treated with DY, DY-B, DYB4 (Fig. 8b), which indicated that the apoptosis was closely related to the inhibition of Wnt/β-catenin signaling pathway by DY, DY-B, DYB4 in HT29 cells.

## Discussion

It has been reported that the abnormal activation of Wnt/β-catenin signaling pathway plays a key role in the development of colorectal cancer, and the inhibitors of Wnt may be an effective treatment for colorectal cancer [1]. In our previous study, we found that the aqueous extract DY of *S. officinalis* strongly inhibited Wnt/β-catenin signaling pathway, so we continued to study the active compounds and the underlying molecular mechanism. In this study, we found that 1,4,6-tri-*O*-galloyl-β-d-glucopyranose (TGG), shows significant inhibition effect on the Wnt signaling pathway (Fig. 1b), which was reported to inhibit human cancer K562, HL-60, HeLa cells in vitro and as positive control of antitumor activity in murine sarcoma S180 tumor-bearing Kunming mice [17]. Further mechanistic study showed that TGG inhibited the Wnt pathway by down-regulating the level of β-catenin and the expression of some Wnt target genes (Dkk1 [20], c-Myc [21], FGF20, NKD-1 [22], Survivin [23]) (Fig. 3c). β-catenin occupies the core position in the Wnt pathway. When the amount of β-catenin increases in cytoplasm, it will enter the nucleus, which leads to many cellular events including proliferation, invasion, metastasis [33]. Moreover, the levels of β-catenin in colon cancer cells are substantially increased compared with normal tissues [34]. Therefore, targeting β-catenin would be a potential way in reversing excessive activation of Wnt pathway in colon cancer cells. However, few of natural compounds have so far been found to target β-catenin. Resveratrol, extracted from Chinese herbal medicine Polygonum cuspidatum, can decrease nuclear localization of β-catenin thus attenuated Wnt/β-catenin signaling, which leads to the inhibition of CRC invasion and metastasis [35]. Alkaloid berberine, as a traditional anti-inflammatory Chinese herbal medicine, can inhibit colon tumor formation through inhibition of Wnt/β-catenin signaling [36]. Wenyang Huazhuo Tongluo formula has anti-fibrosis effect in Bleomycin-induced SSc mouse model by inhibiting Wnt/β-catenin signaling pathway [37]. Thus, our study provides a valuable prototype for effective therapeutic agents in tumor treatment by inhibiting Wnt pathway.

In the next generation sequencing results, the GO terms analysis revealed that the DEGs caused by these drugs were mainly involved in biological process, cell composition and molecular function, which were related with cell metabolism, apoptosis and proliferation (Additional file 2: Figure S10). Specially, from the top 10 functions of GO terms, we found that the positive regulation of apoptotic process [31] was highly significant in DY, DY-B, DYB4 groups (Additional file 2: Figure S4), which may be potentially associated with the treatment of colorectal cancer. Additionally, based on the KEGG analysis of DEGs enrichment, we found that the over-representation of the pathway in cancer and the PI3K/Akt signaling pathway was observed in all groups (Additional file 2: Figure S11). PI3K/Akt pathway was closely related to Wnt pathway in tumor cell migration [38].

In terms of Wnt pathway, the mRNA level of Wnt9a, Apc were up-regulated in DY, DY-B, DYB4 groups. Wnt9a [29] as the non-canonical Wnt pathway ligand, can inhibit proliferation and increase apoptosis by suppressing β-catenin. And the Apc [10] also can promote the degradation of β-catenin by forming the so-called destruction complex with Gsk3β and Axin. In addition, DY also up-regulated the expression of Dkk2. DYB4 up-regulated the expression of Gsk3β (Fig. 6). The up-regulation of Wnt9a, Apc, Dkk2 [30], Gsk3β [10] was relevant to the inhibition of Wnt signaling pathway. These results help to explain the molecular mechanism of *S. officinalis* on Wnt.

Moreover, most studies have reported that the cell apoptosis can be induced with the inhibition of Wnt signaling pathway by targeting on the Wnt related genes, such as Apc, Gsk3β, c-Myc, NKD-1, Survivin, Wnt9a and so on. Our previous results on the level of caspase3, PARP, Bax and Bcl-2 [31] were used to evaluate the apoptosis of HT29 treated with DY, DY-B, DYB4. The caspase3, PARP and the ratio of Bax/Bcl-2 were up-regulated (Fig. 7) and HT29 cells were blocked in G1/G0 phase (Fig. 8), suggesting that the intrinsic apoptotic activity was activated under the suppression of Wnt/β-catenin signaling pathway.

## Conclusion

Our study indicates that DY, DY-B and DYB4 from *S. officinalis* effectively inhibit the Wnt/β-catenin signaling pathway. The inhibition activity was possibly mediated by the down-regulation of β-catenin or the Wnt-related genes. Additionally, DY, DY-B and DYB4 up-regulate the level of caspase3, PARP and the ratio of Bax/Bcl-2, and induced the apoptosis of HT29 cells. Taken together, our findings demonstrate that DYB4 (TGG), is a potential anti-Wnt agent for the treatment of colorectal cancer.

## Additional files

**Additional file 1.** Minimum standards of reporting checklist.

**Additional file 2.** Additional Figures and Table.

## Data Availability

All data generated or analyzed during this study are included in this article and its additional files.

## Abbreviations

DYB4: 1,4,6-Tri-*O*-galloyl-β-d-glucopyranose (TGG)
DY: aqueous extract of *Sanguisorba officinalis*
DY-B: 40% ethanol elution from *Sanguisorba officinalis* with D101 macroporous absorbent resin column

## References

[1] Ferrarelli LK (2017). Treating WNT-driven colorectal cancer. Science.

[2] Galamb O, Kalmar A, Peterfia B, Csabai I, Bodor A, Ribli D, Krenacs T, Patai AV, Wichmann B, Bartak BK (2016). Aberrant DNA methylation of WNT pathway genes in the development and progression of CIMP-negative colorectal cancer. Epigenetics.

[3] Rennoll S, Yochum G (2015). Regulation of MYC gene expression by aberrant Wnt/beta-catenin signaling in colorectal cancer. World J Biol Chem.

[4] Bendelsmith CR, Skrypek MM, Patel SR, Pond DA, Linabery AM, Bendel AE (2018). Multiple pilomatrixomas in a survivor of WNT-activated medulloblastoma leading to the discovery of a germline APC mutation and the diagnosis of familial adenomatous polyposis. Pediatr Blood Cancer..

[5] Cui J, Zhou X, Liu Y, Tang Z, Romeih M (2003). Wnt signaling in hepatocellular carcinoma: analysis of mutation and expression of beta-catenin, T-cell factor-4 and glycogen synthase kinase 3-beta genes. J Gastroenterol Hepatol.

[6] Cersosimo RJ (2013). Management of advanced colorectal cancer, Part 1. Am J Health Syst Pharm.

[7] Heidelberger C, Chaudhuri NK, Danneberg P, Mooren D, Griesbach L, Duschinsky R, Schnitzer RJ, Pleven E, Scheiner J (1957). Fluorinated pyrimidines, a new class of tumour-inhibitory compounds. Nature.

[8] Qin W, Zheng Y, Qian BZ, Zhao M (2017). Prostate cancer stem cells and nanotechnology: a focus on Wnt signaling. Front Pharmacol.

[9] Berwick DC, Harvey K (2014). The regulation and deregulation of Wnt signaling by PARK genes in health and disease. J Mol Cell Biol.

[10] Pronobis MI, Rusan NM, Peifer M (2015). A novel GSK3-regulated APC: Axin interaction regulates Wnt signaling by driving a catalytic cycle of efficient beta catenin destruction. eLife.

[11] Voloshanenko O, Gmach P, Winter J, Kranz D, Boutros M (2017). Mapping of Wnt-Frizzled interactions by multiplex CRISPR targeting of receptor gene families. FASEB J.

[12] MacDonald BT, He X (2012). Frizzled and LRP5/6 receptors for Wnt/beta-catenin signaling. Cold Spring Harb Perspect Biol..

[13] Shimizu N, Kawakami K, Ishitani T (2012). Visualization and exploration of Tcf/Lef function using a highly responsive Wnt/beta-catenin signaling-reporter transgenic zebrafish. Dev Biol.

[14] Jang E, Inn KS, Jang YP, Lee KT, Lee JH (2018). Phytotherapeutic activities of *Sanguisorba officinalis* and its chemical constituents: a review. Am J Chin Med.

[15] Liu MP, Liao M, Dai C, Chen JF, Yang CJ, Liu M, Chen ZG, Yao MC (2016). *Sanguisorba officinalis* L. synergistically enhanced 5-fluorouracil cytotoxicity in colorectal cancer cells by promoting a reactive oxygen species-mediated, mitochondria-caspase-dependent apoptotic pathway. Sci Rep.

[16] Liu MP, Li W, Dai C, Lam CWK, Li Z, Chen JF, Chen ZG, Zhang W, Yao MC (2018). Aqueous extract of *Sanguisorba officinalis* blocks the Wnt/-catenin signaling pathway in colorectal cancer cells. Rsc Adv.

[17] Li CW, Dong HJ, Cui CB (2015). The synthesis and antitumor activity of twelve galloyl glucosides. Molecules.

[18] Polakis P (2002). Casein kinase 1: a Wnt’er of disconnect. Curr Biol.

[19] Dakeng S, Duangmano S, Jiratchariyakul W, U‐Pratya Y, Bögler O, Patmasiriwat P (2012). Inhibition of Wnt signaling by cucurbitacin B in breast cancer cells: reduction of Wnt-associated proteins and reduced translocation of galectin-3-mediated beta-catenin to the nucleus. J Cell Biochem.

[20] Hirata H, Hinoda Y, Nakajima K, Kawamoto K, Kikuno N, Ueno K, Yamamura S, Zaman MS, Khatri G, Chen Y (2011). Wnt antagonist DKK1 acts as a tumor suppressor gene that induces apoptosis and inhibits proliferation in human renal cell carcinoma. Int J Cancer.

[21] Shi L, Wu YX, Yu JH, Chen X, Luo XJ, Yin YR (2017). Research of the relationship between beta-catenin and c-myc-mediated Wnt pathway and laterally spreading tumors occurrence. Eur Rev Med Pharmacol Sci.

[22] Stancikova J, Krausova M, Kolar M, Fafilek B, Svec J, Sedlacek R, Neroldova M, Dobes J, Horazna M, Janeckova L (2015). NKD1 marks intestinal and liver tumors linked to aberrant Wnt signaling. Cell Signal.

[23] Ho YS, Tsai WH, Lin FC, Huang WP, Lin LC, Wu SM, Liu YR, Chen WP (2016). Cardioprotective actions of TGFbetaRI inhibition through stimulating autocrine/paracrine of survivin and inhibiting Wnt in Cardiac progenitors. Stem Cells.

[24] Kandimalla R, Linnekamp JF, van Hooff S, Castells A, Llor X, Andreu M, Jover R, Goel A, Medema JP (2017). Methylation of WNT target genes AXIN2 and DKK1 as robust biomarkers for recurrence prediction in stage II colon cancer. Oncogenesis.

[25] Zhou F, Gong K, van Laar T, Gong Y, Zhang L (2011). Wnt/beta-catenin signal pathway stabilizes APP intracellular domain (AICD) and promotes its transcriptional activity. Biochem Biophys Res Commun.

[26] Gordon MD, Nusse R (2006). Wnt signaling: multiple pathways, multiple receptors, and multiple transcription factors. J Biol Chem.

[27] Chen CL, Yang JX, James IOA, Zhang HY, Besner GE (2014). Heparin-binding epidermal growth factor-like growth factor restores Wnt/beta-catenin signaling in intestinal stem cells exposed to ischemia/reperfusion injury. Surgery.

[28] Alvania RS, Chen X, Ginty DD (2006). Calcium signals control Wnt-dependent dendrite growth. Neuron.

[29] Rochard L, Monica SD, Ling IT, Kong Y, Roberson S, Harland R, Halpern M, Liao EC (2016). Roles of Wnt pathway genes wls, wnt9a, wnt5b, frzb and gpc4 in regulating convergent-extension during zebrafish palate morphogenesis. Development.

[30] Olivares-Navarrete R, Hyzy S, Wieland M, Boyan BD, Schwartz Z (2010). The roles of Wnt signaling modulators Dickkopf-1 (Dkk1) and Dickkopf-2 (Dkk2) and cell maturation state in osteogenesis on microstructured titanium surfaces. Biomaterials.

[31] Hu Y, Yu K, Wang G, Zhang D, Shi C, Ding Y, Hong D, Zhang D, He H, Sun L (2018). Lanatoside C inhibits cell proliferation and induces apoptosis through attenuating Wnt/beta-catenin/c-Myc signaling pathway in human gastric cancer cell. Biochem Pharmacol.

[32] Ali I, Medegan B, Braun DP (2016). Wnt9A induction linked to suppression of human colorectal cancer cell proliferation. Int J Mol Sci.

[33] Bai J, Luo X (2018). 5-Hydroxy-4′-nitro-7-propionyloxy-genistein inhibited invasion and metastasis via inactivating Wnt/b-catenin signal pathway in human endometrial carcinoma ji endometrial cells. Med Sci Monit.

[34] Rice PL, Kelloff J, Sullivan H, Driggers LJ, Beard KS, Kuwada S, Piazza G, Ahnen DJ (2003). Sulindac metabolites induce caspase- and proteasome-dependent degradation of beta-catenin protein in human colon cancer cells. Mol Cancer Ther.

[35] Ji Q, Liu X, Fu X, Zhang L, Sui H, Zhou L, Sun J, Cai J, Qin J, Ren J (2013). Resveratrol inhibits invasion and metastasis of colorectal cancer cells via MALAT1 mediated Wnt/beta-catenin signal pathway. PLoS ONE.

[36] Zhang J, Cao H, Zhang B, Cao H, Xu X, Ruan H, Yi T, Tan L, Qu R, Song G (2013). Berberine potently attenuates intestinal polyps growth in ApcMin mice and familial adenomatous polyposis patients through inhibition of Wnt signalling. J Cell Mol Med.

[37] Wang Q, Zang W, Han L, Yang L, Ye S, Ouyang J, Zhang C, Bi Y, Zhang C, Bian H (2018). Wenyang Huazhuo Tongluo formula inhibits fibrosis via suppressing Wnt/beta-catenin signaling pathway in a Bleomycin-induced systemic sclerosis mouse model. Chin Med.

[38] Liu M, Gao X, Liu CL (2018). Increased expression of lncRNA FTH1P3 promotes oral squamous cell carcinoma cells migration and invasion by enhancing PI3K/Akt/GSK3b/Wnt/beta-catenin signaling. Eur Rev Med Pharmacol Sci.

