# Supplementary material for: A tannin compound from Sanguisorba officinalis blocks Wnt/β-catenin signaling pathway and induces apoptosis of colorectal cancer cells
Source: Chin Med. 2019 May 31;14:22. doi: 10.1186/s13020-019-0244-y (PMC6544925; doi:10.1186/s13020-019-0244-y)
Supplement: Supplementary file 2 — Additional file 2. Additional Figures and Table. [file 13020_2019_244_MOESM2_ESM.docx]

***Additional material:***


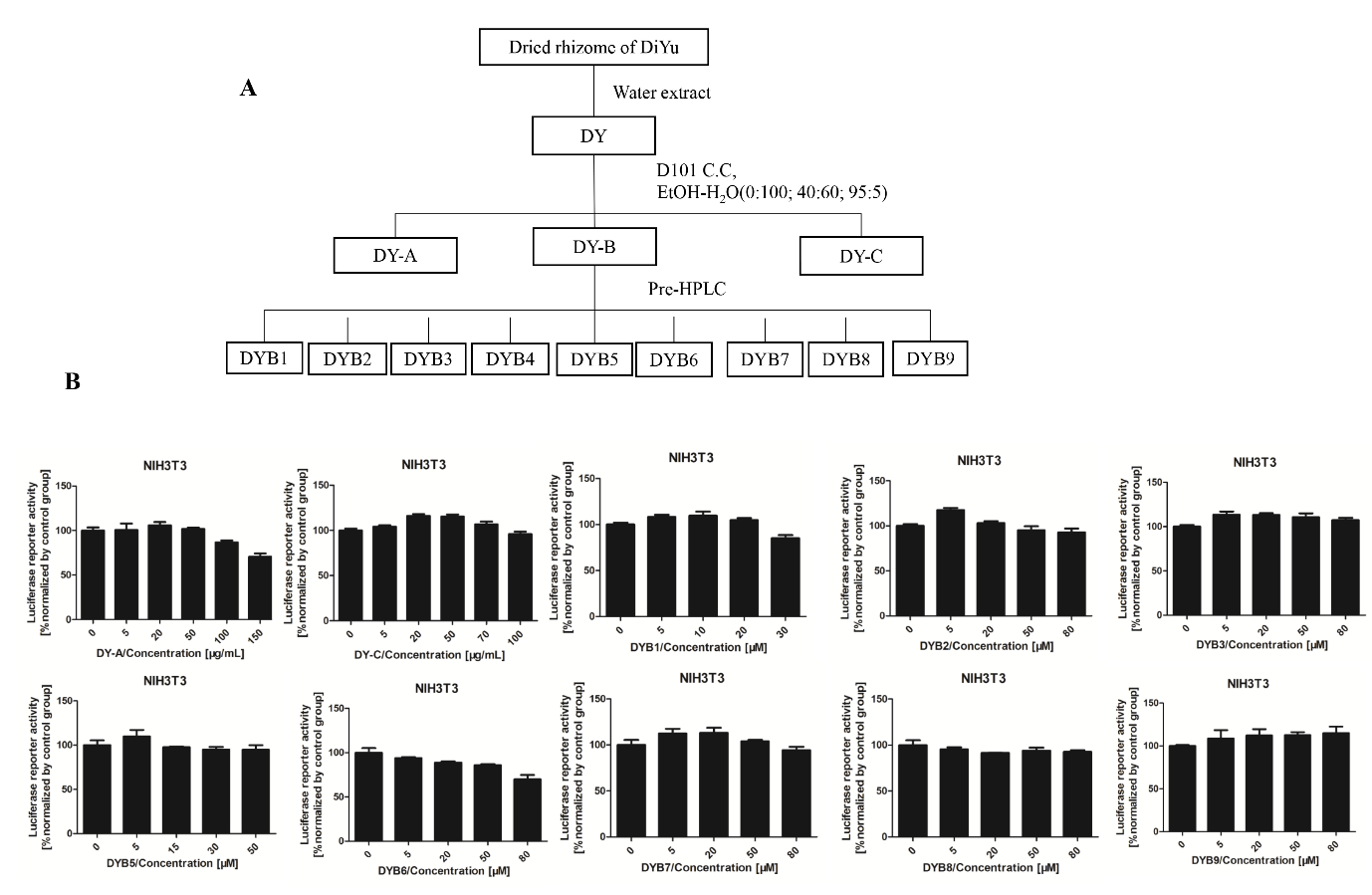


***Figure S1*. Luciferase reporter assay of the components of** ***Sanguisorba officinalis*. (**A) the preparation process for different components. (B) the inhibitory effect on Wnt signaling pathway of these components.


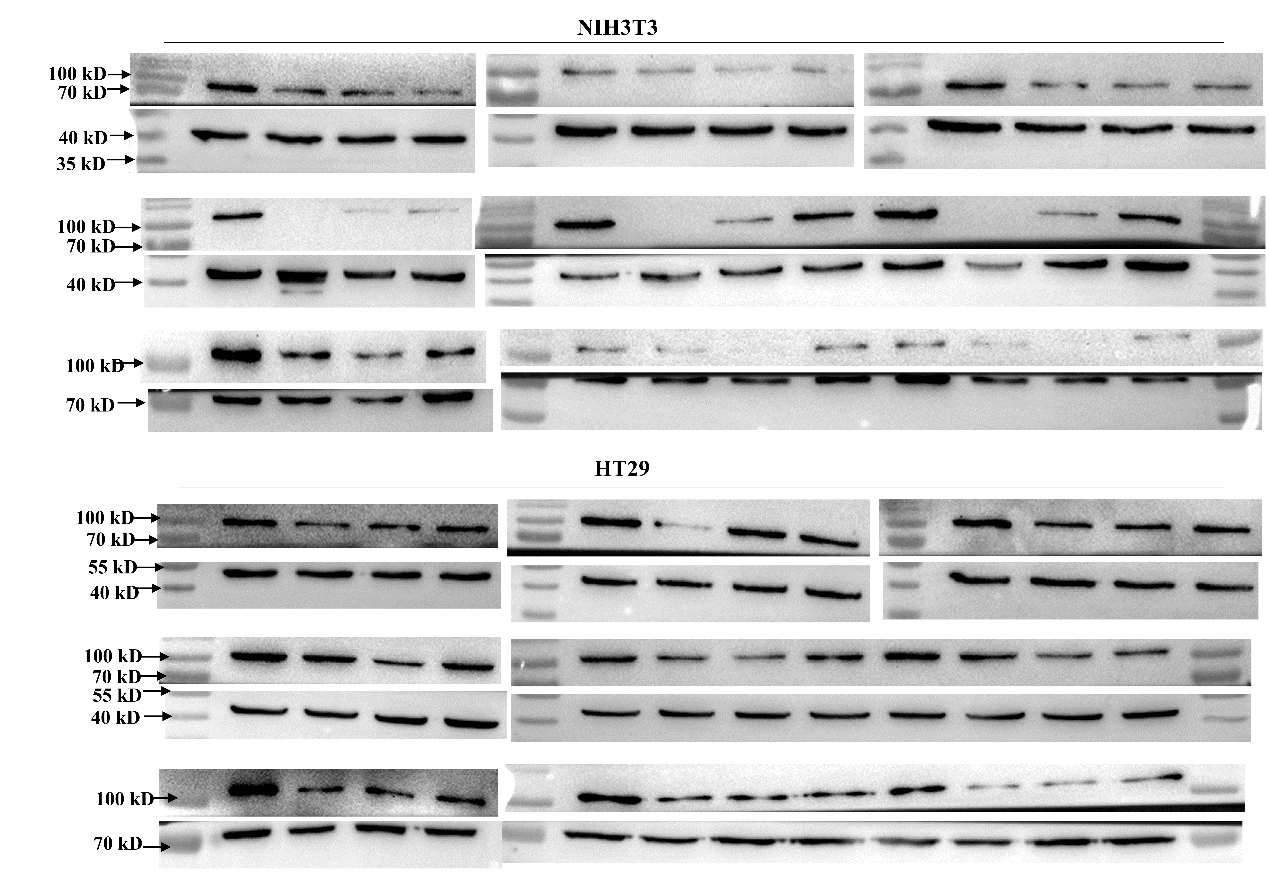


***Figure S2.* Western blots with molecular size markers for the indicated protein in the manuscript.** These figures are the blots of β-catenin and actin in NIH3T3 and HT29 cells from three replicated experiments, respectively. The blots of each protein in the control, DY, DY-B and DYB4 groups are presented from left to right, and the bands of β-catenin in Total protein, Cytoplasmic Protein and nucleoprotein are presented from top to bottom in each cell line.

**
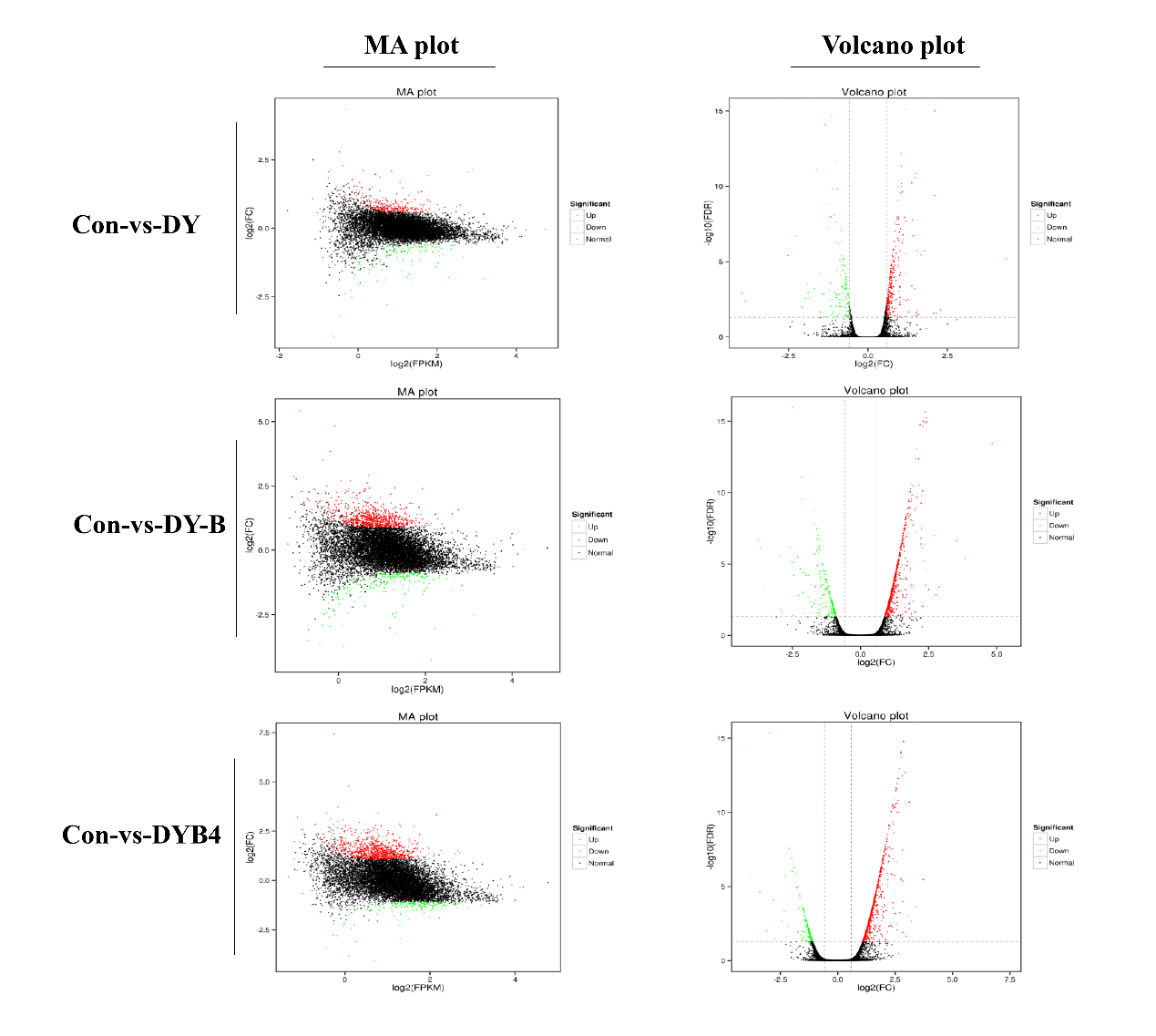
**

***Figure S3.* Integral distribution of the DEGs of HT29 cells.** The MA graphs and volcano plots of DEGs induced by DY (40 μg/ml), DY-B (40 μg/ml) and DYB4 (40 μM). FC, fold change; FDR, False Discovery Rate; FPKM, Fragment Per Kilobase of exon model per Million mapped reads. The up-regulated, down-regulated and normal genes are respectively plotted as red, green and black dots. DESeq was used to perform the DEGs analysis.


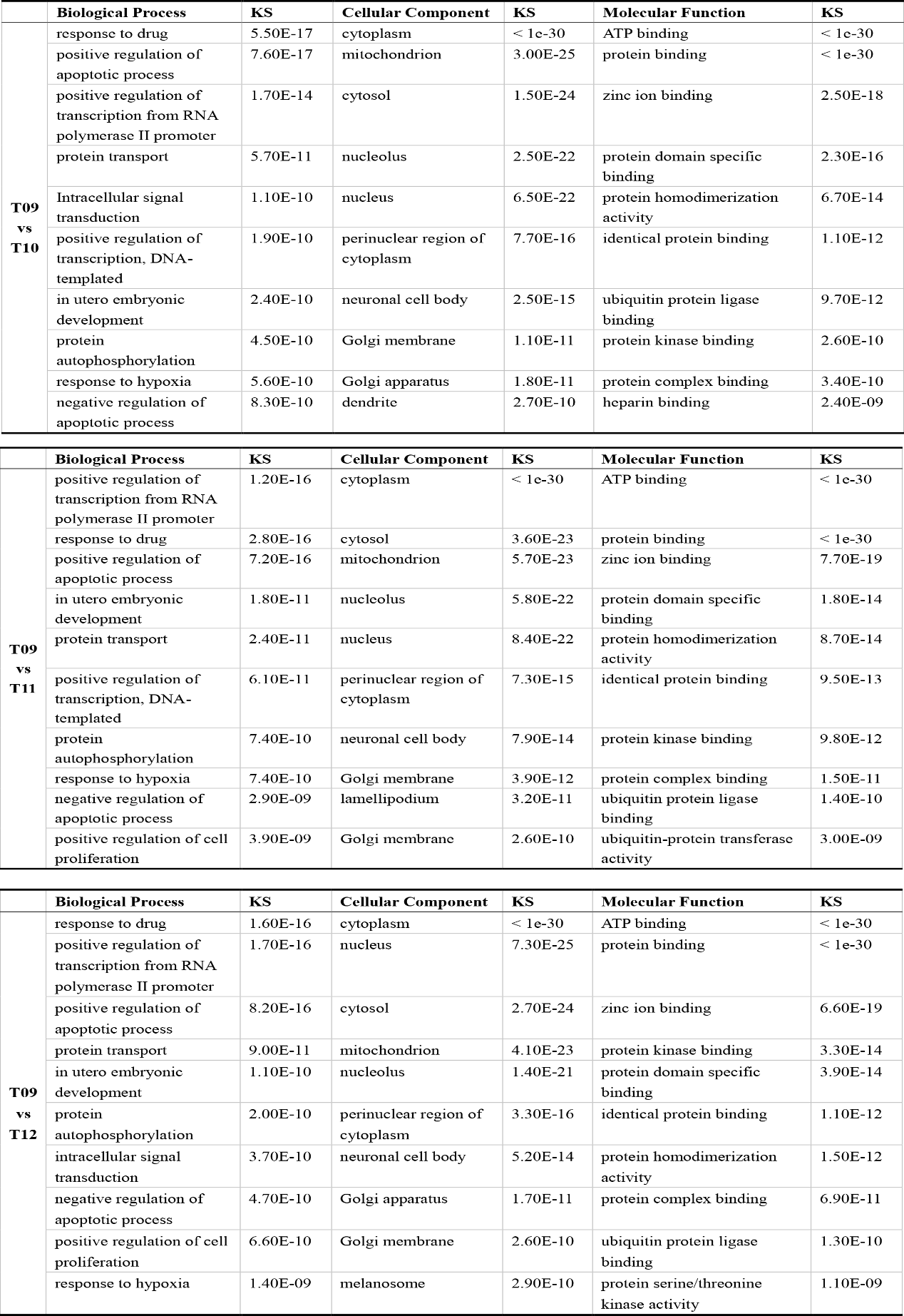


***Figure S4.* The top 10 secondary functions analysis of GO enrichment in Biological Process, Cellular Component, Molecular Function.** KS represented the significant difference of the GO enrichment. The significance of the statistics difference increased with KS value. T09, T10, T11, T12 respectively represented the control, DY, DY-B, DYB4 groups.

**
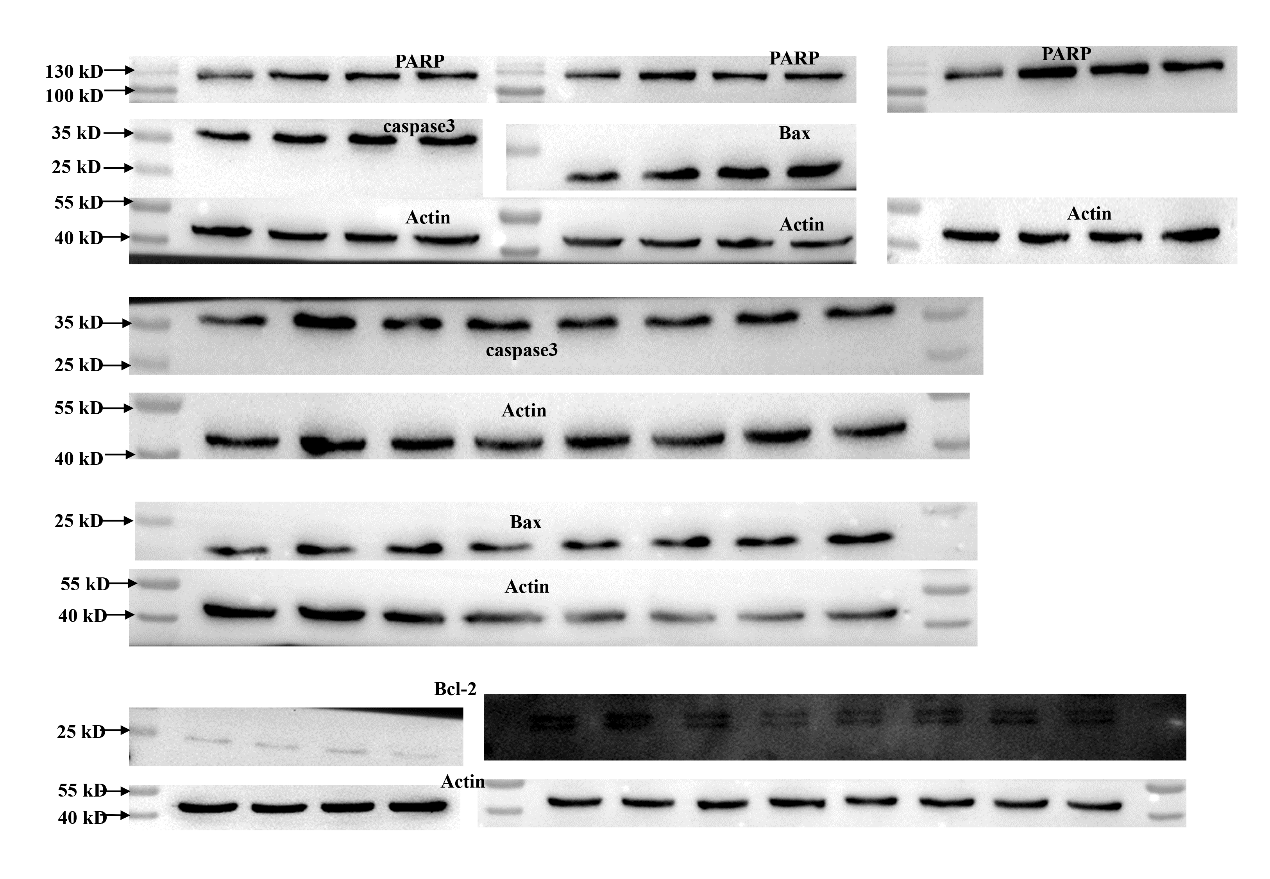
**

***Figure S5.* Western blots with molecular size markers for the indicated protein in the manuscript.** These figures are the blots of PARP, caspase3, Bax, Bcl-2 and actin in HT29 cells from three replicated experiments, respectively. The blots of each protein in the control, DY, DY-B and DYB4 groups are presented from left to right.


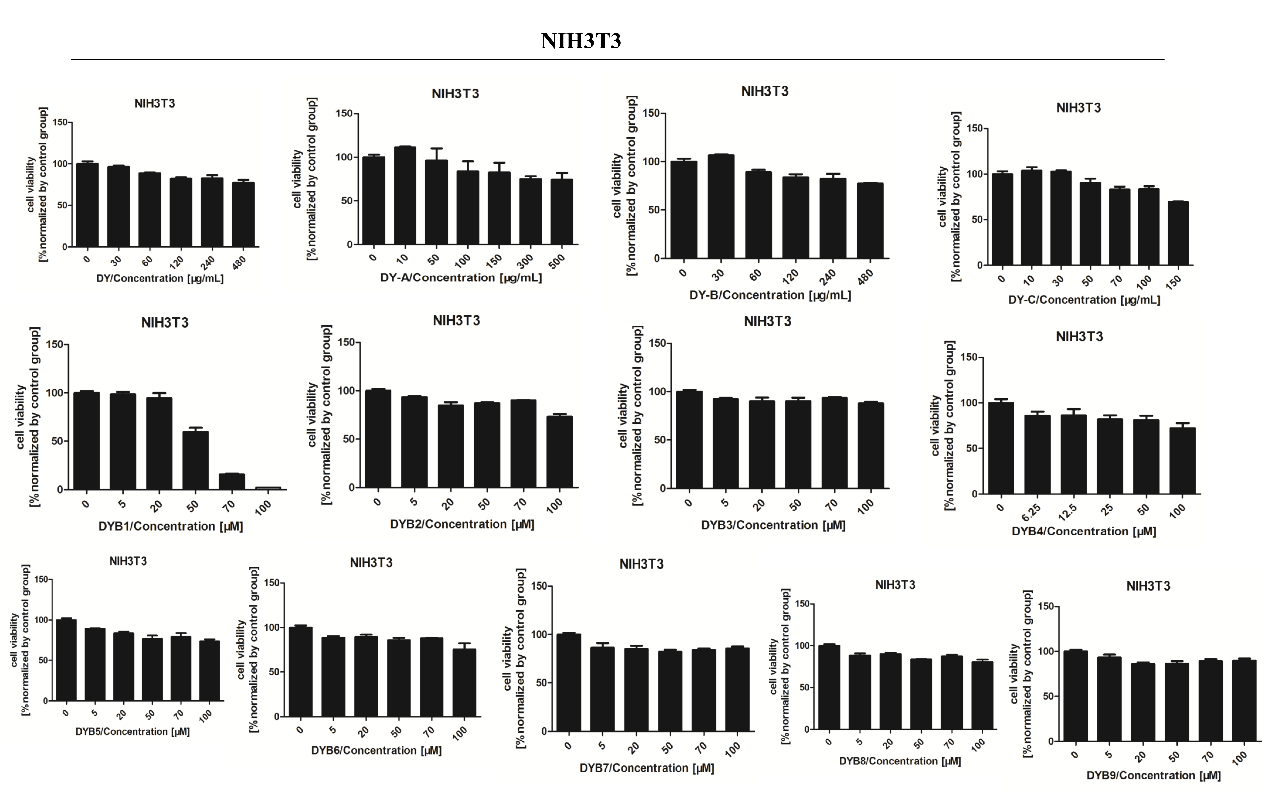


***Figure S6.* Cell viability assays of *Sanguisorba officinalis* and its components in NIH3T3 cells.**


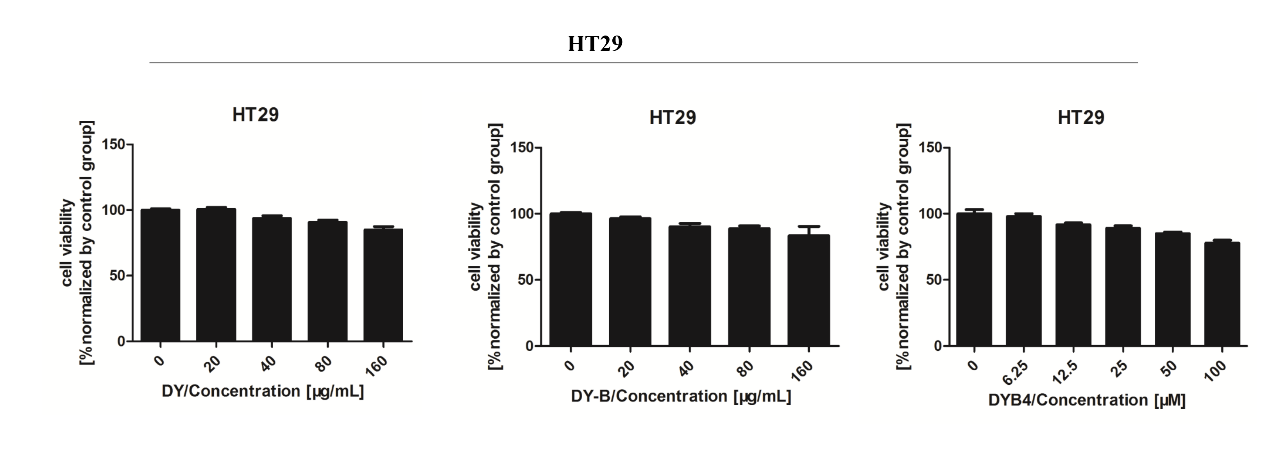


***Figure S7.* Cell viability assays of *Sanguisorba officinalis* and its components in HT29 cells.**

***Figure S8.* The HPLC chromatogram of TGG.**

**
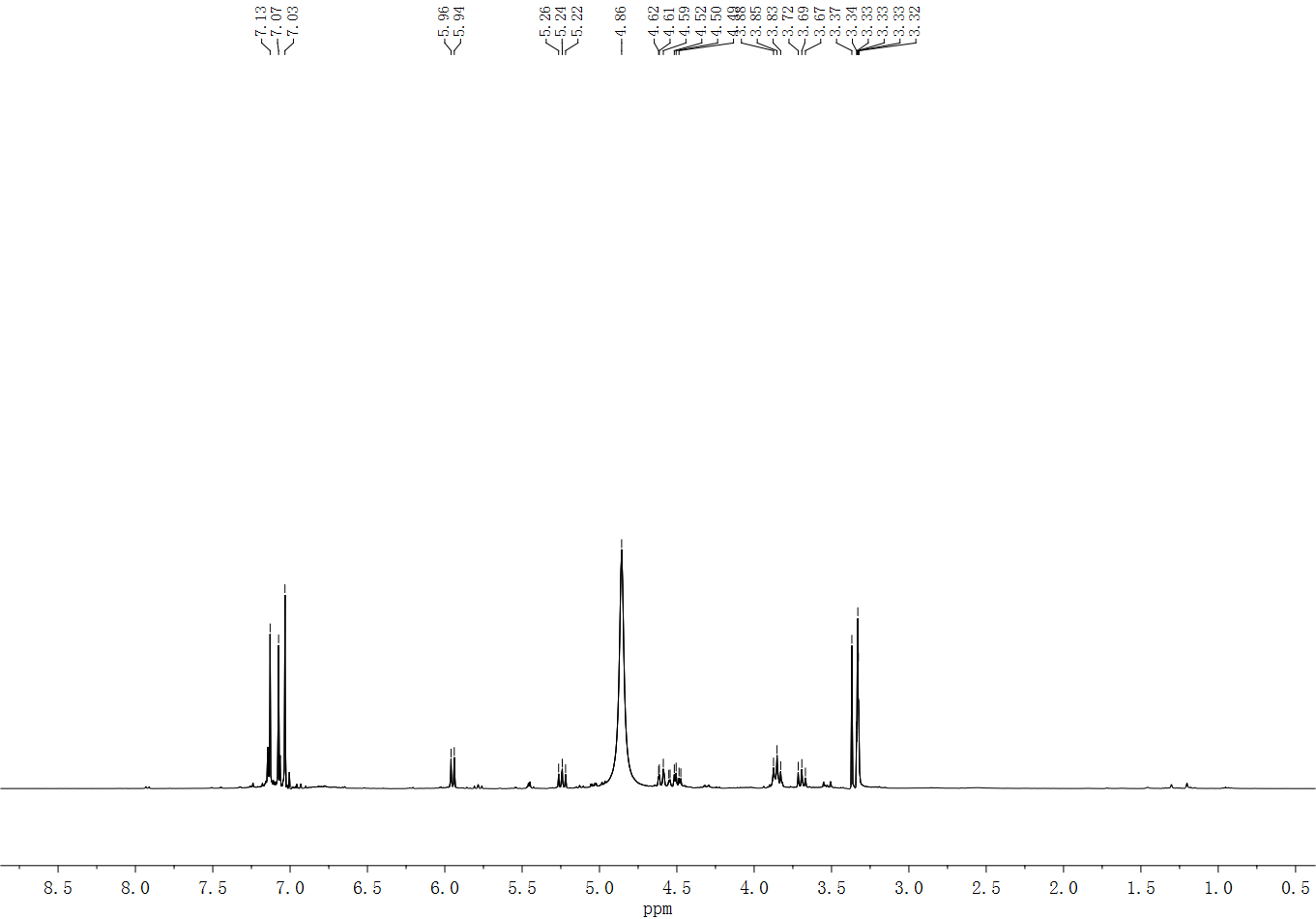
**

***Figure s9.*** **The ^1^H-NMR information of DYB4**


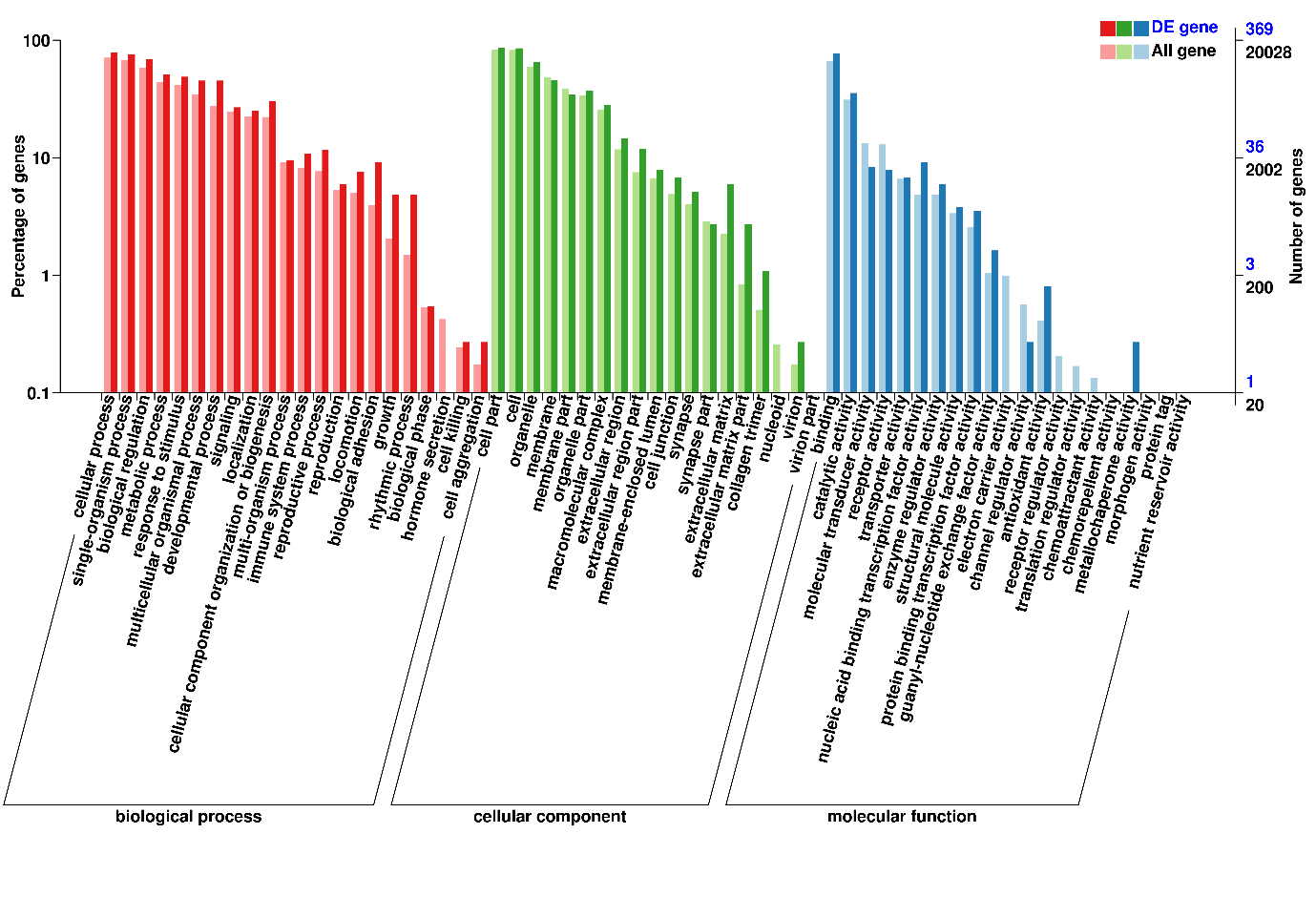


**Con-vs-DY**


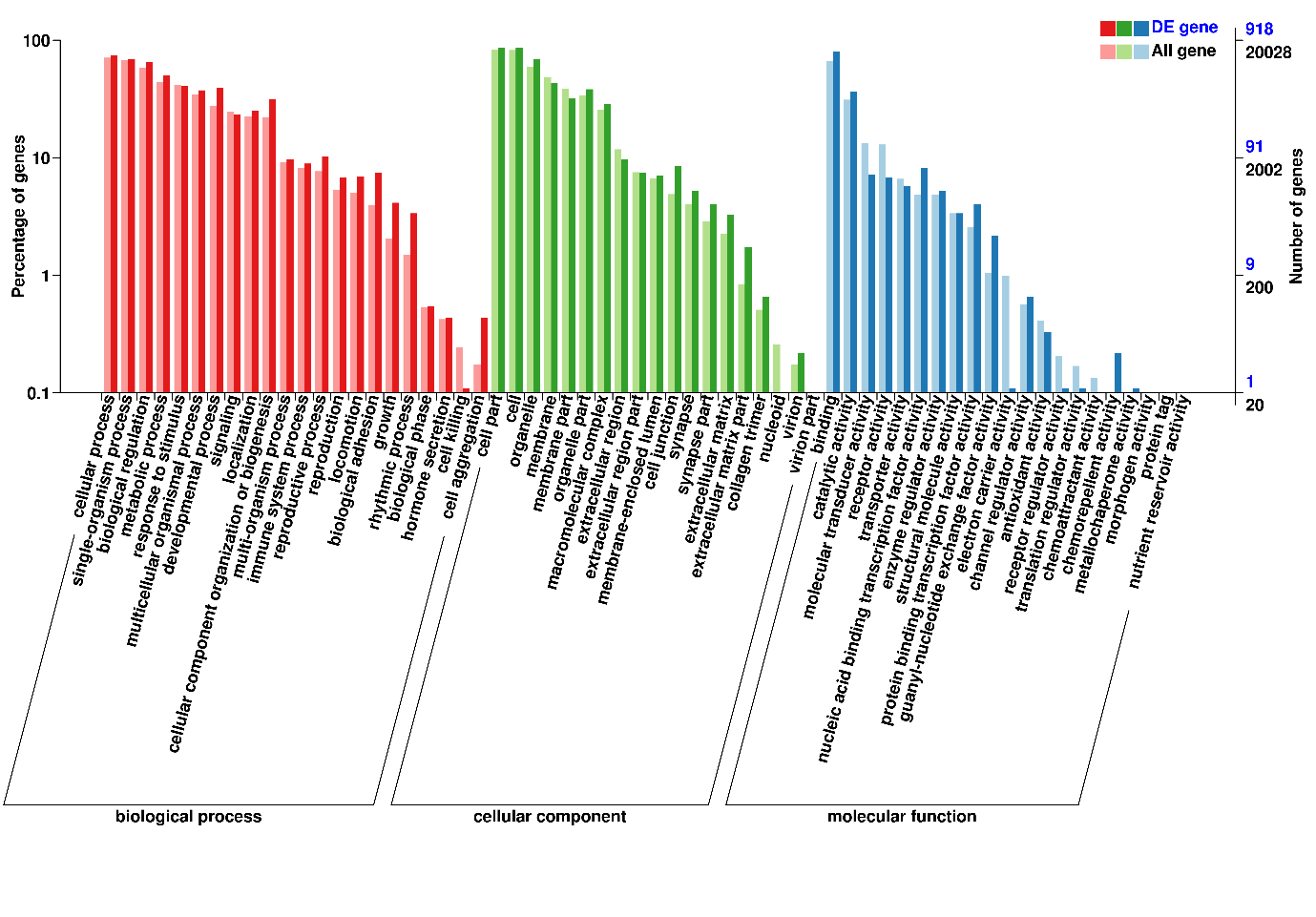


**Con-vs-DY-B**


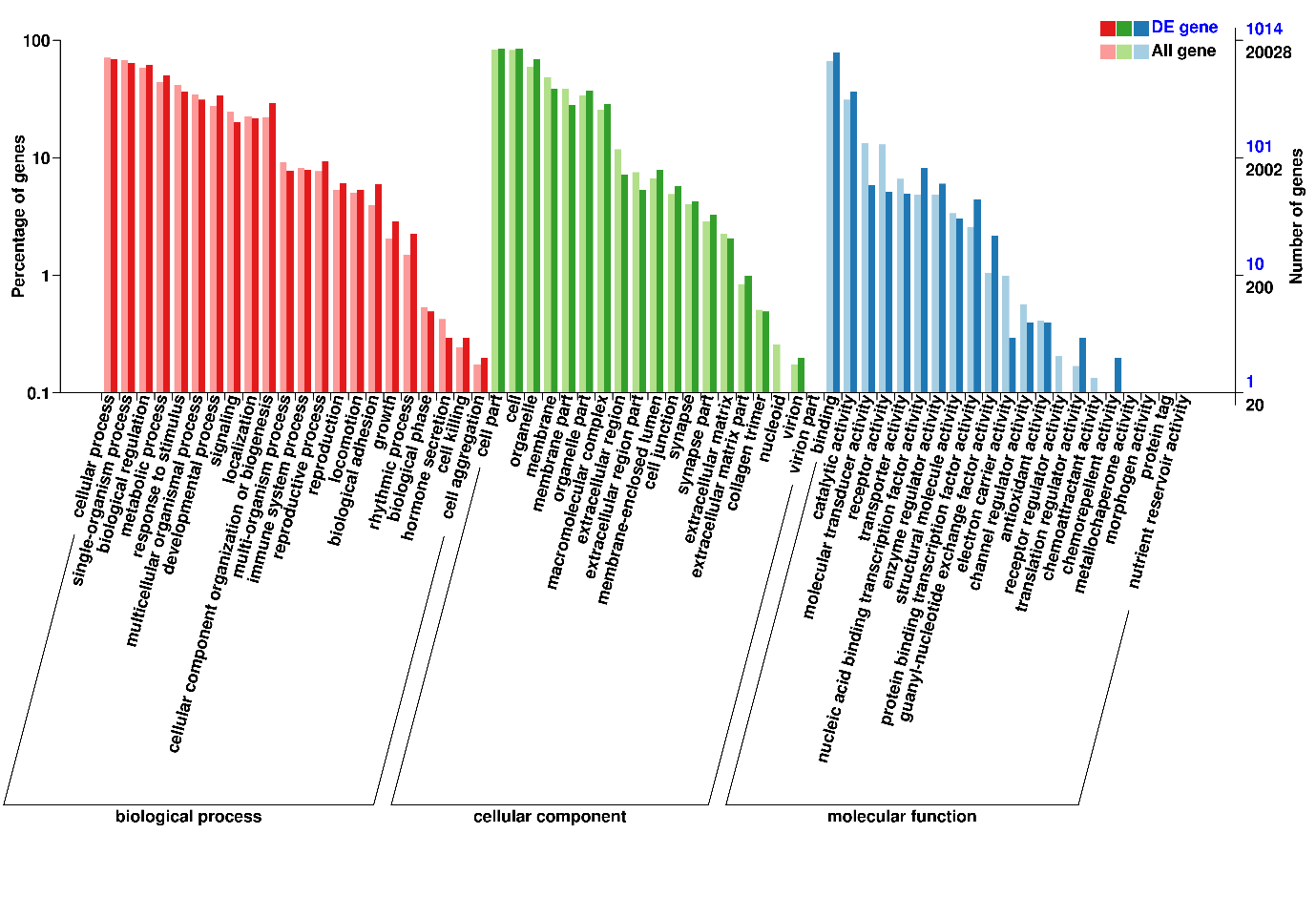


**Con-vs-DYB4**

***Figure s10.* GO classification of the DEGs.** X-coordinate shows the classifications of the GO items, and the left and right sides of y-coordinate present the percentage and numbers of genes. The secondary functions with significant differences (DEGs vs. All genes) are regarded as the potential functions accounting for the enrichments of DEGs.


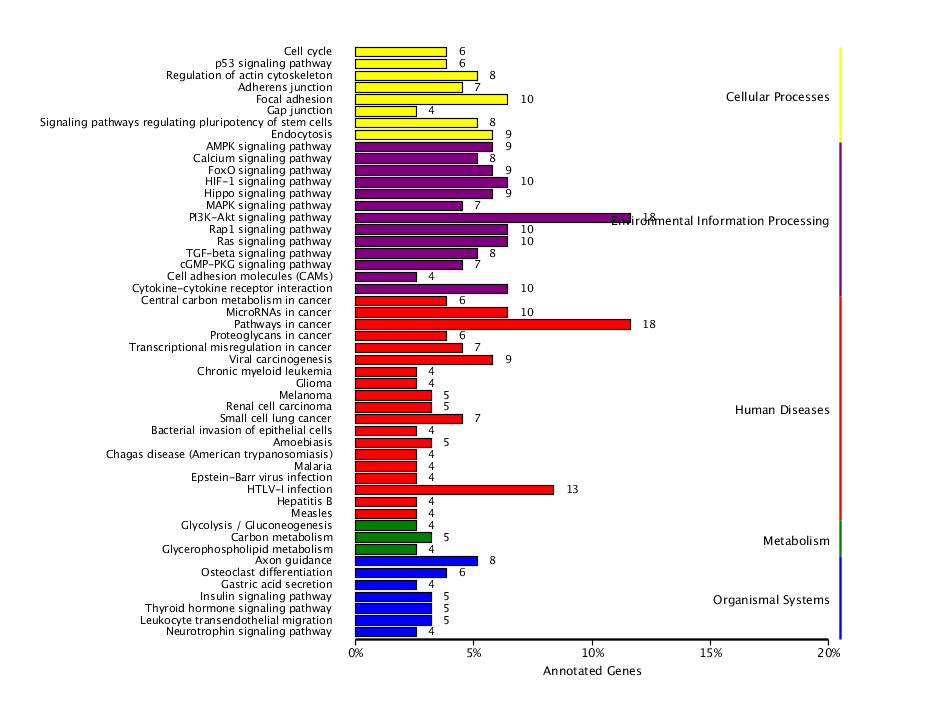


**Con-vs-DY**


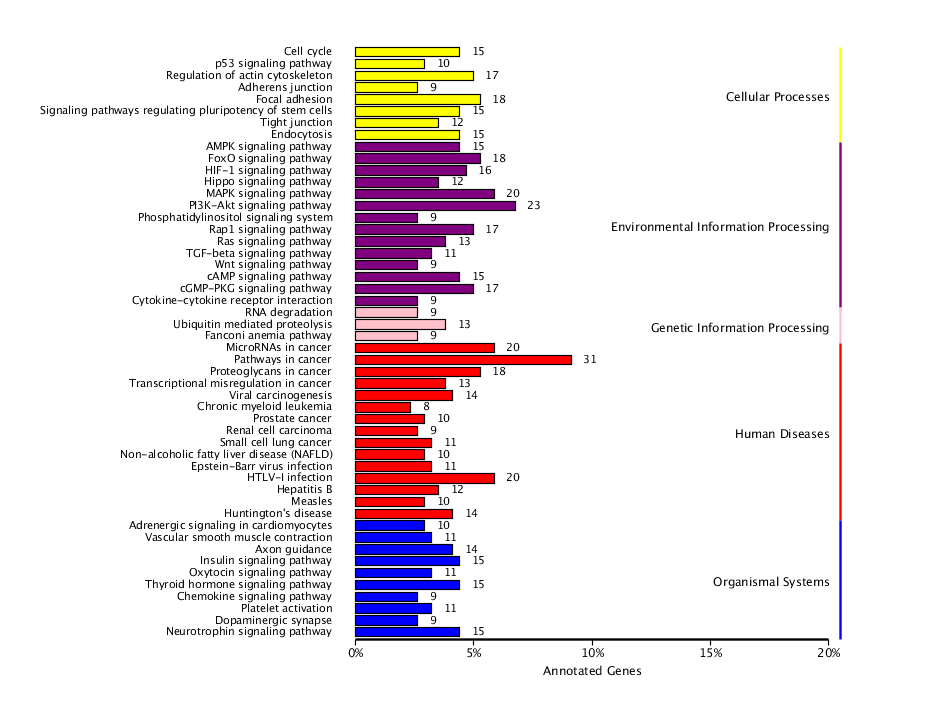

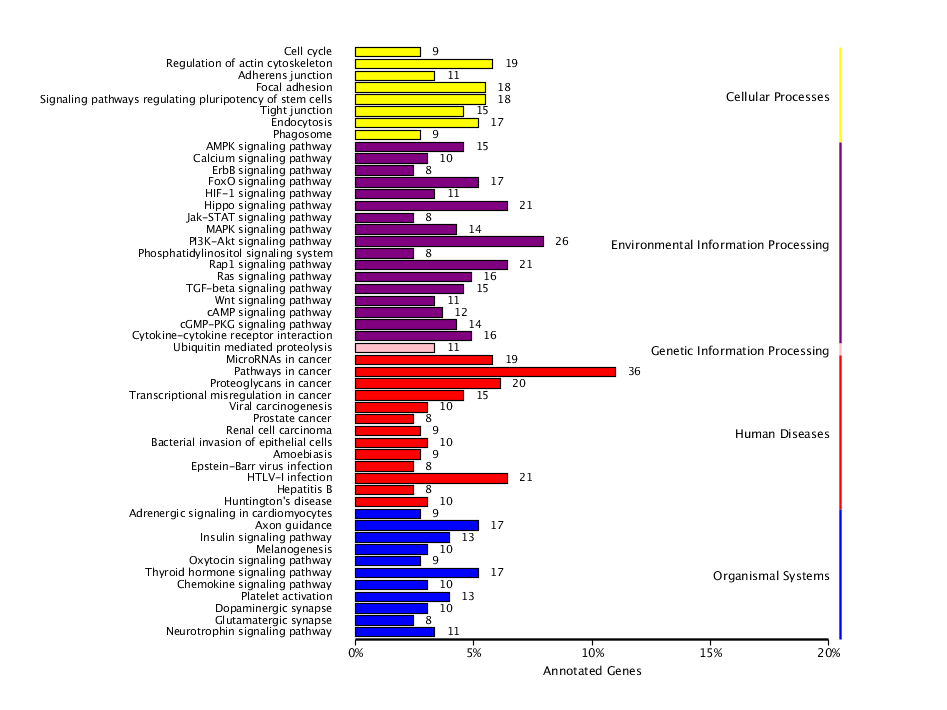


**Con-vs-DYB4**

**Con-vs-DY-B**


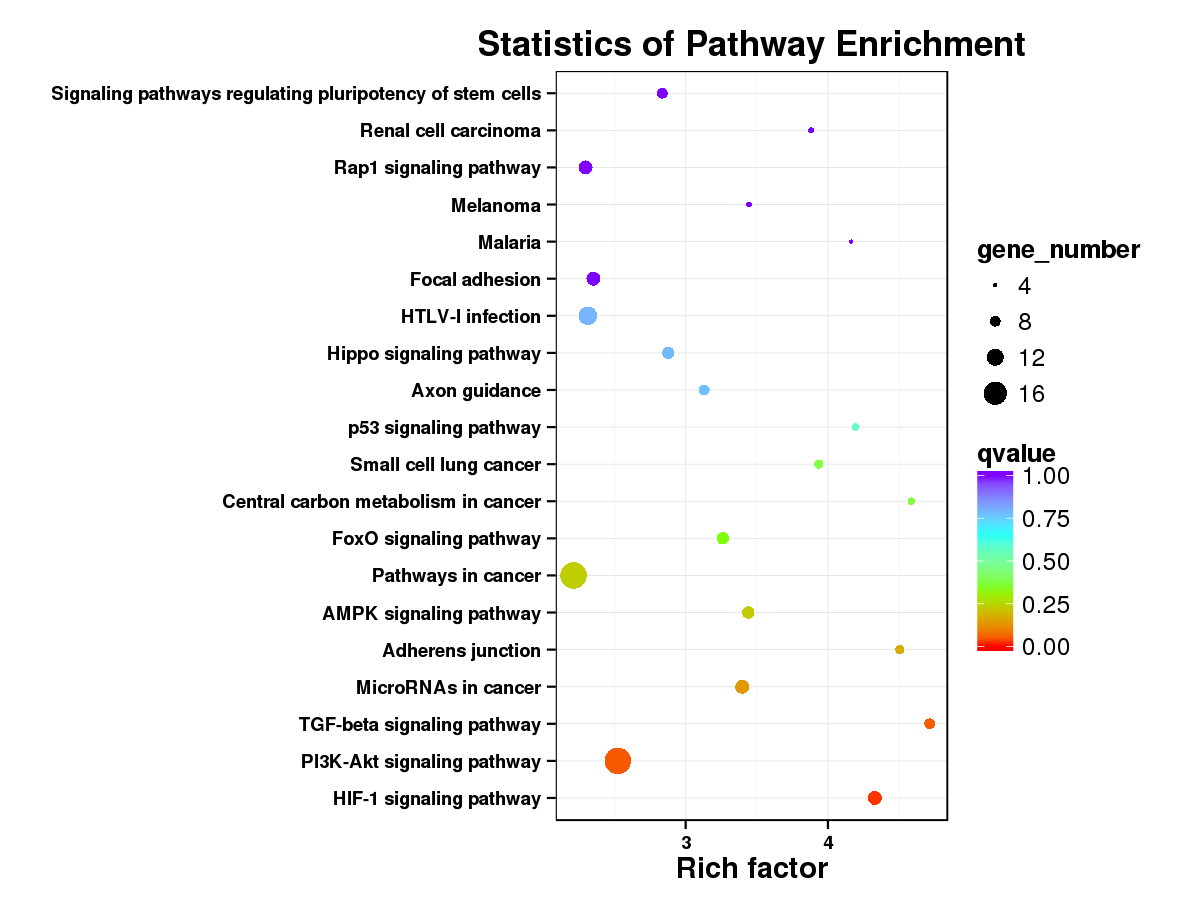


**Con-vs-DY**


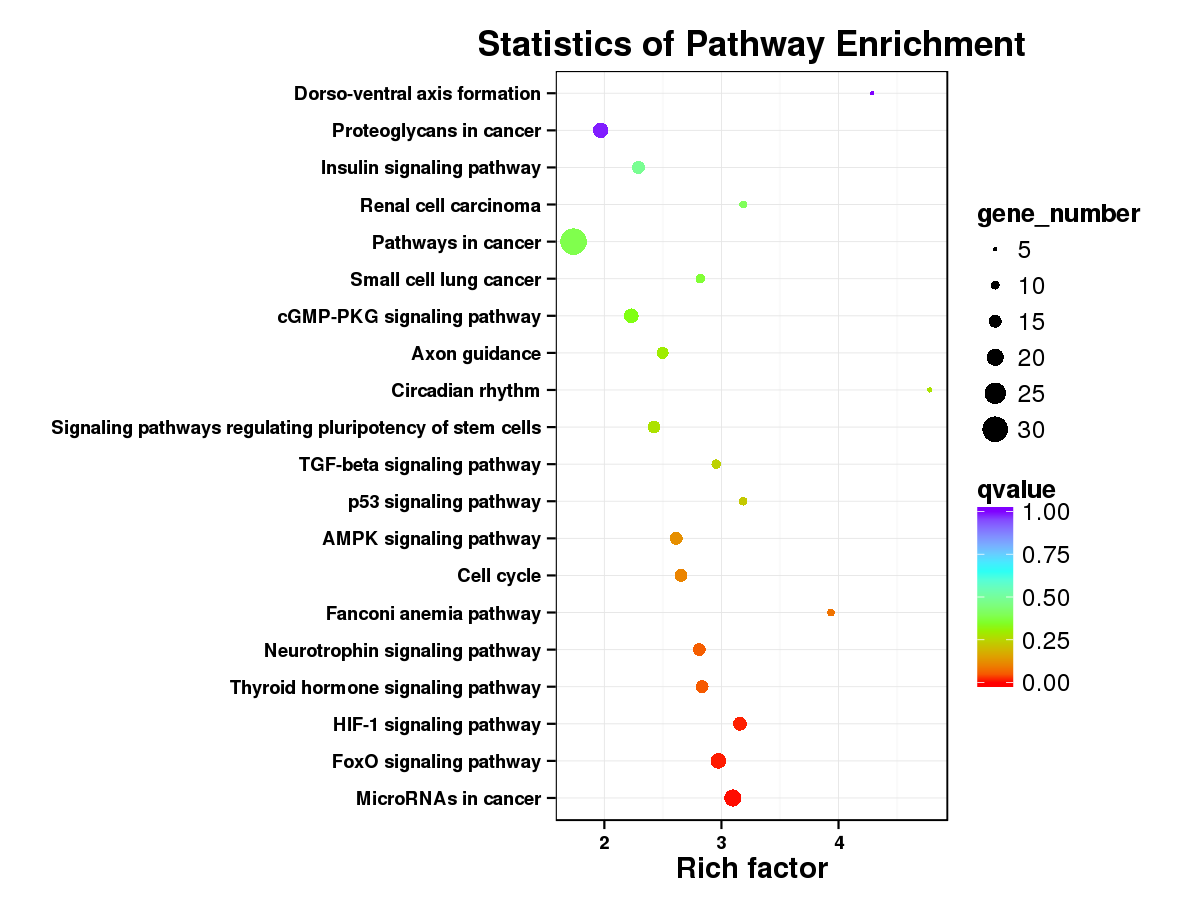

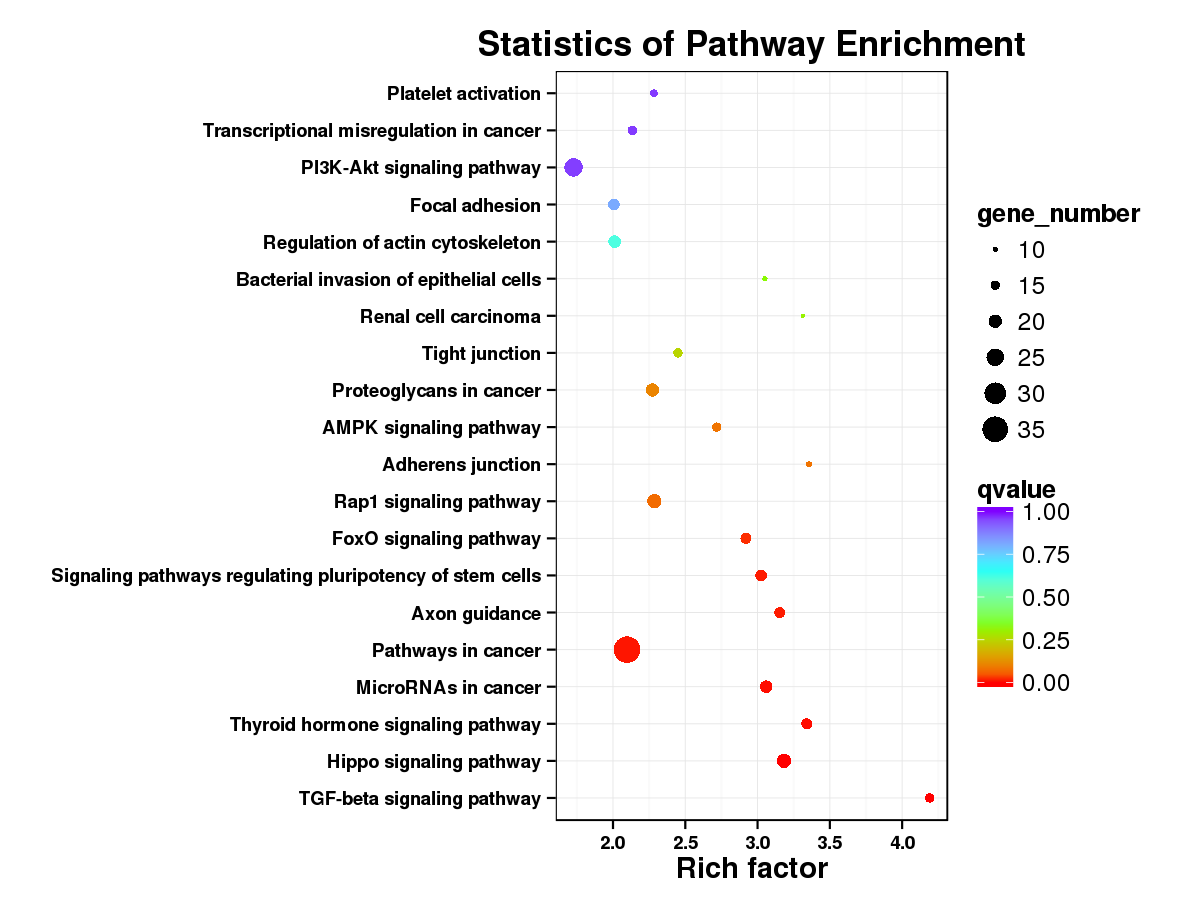


**Con-vs-DY-B**

**Con-vs-DYB4**

***Figure s11.* KEGG classification and enrichment analysis of the DEGs.** In the KEGG classification, X-coordinate shows the number and percentage of the genes annotated in the indicated pathway, while the y-coordinate presents the names of the annotated pathways. In KEGG enrichment analysis, X-coordinate showed the Rich Factor (RF), and the enrichment extent of DEGs in certain pathways enhances with the RF values. Per circle represented a KEGG pathway. The colors and sizes of the circles respectively indicated the P values and gene numbers.

***Table s1.* The sequences of genes primers.**

| Gene primer | Forward | Reverse |
| --- | --- | --- |
| GAPDH | AGGTCGGAGTCAACGGATTTG | TGTAAACCATGTAGTTGAGGTCA |
| Axin2 | AGGCTAGCTGAGGTGT | AGGCTTGGATTGGAGAA |
| Dkk1 | CTGCAAAAATGGAATATGTGT | CTTCTTGTCCTTTGGTGTGA |
| c-Myc | CCACACATCAGCACAACTACG | CCGCAACAAGTCCTCTTCAG |
| FGF20 | ATTCATCAGTGTGGCAGTGG | GCTCCCTAAAGATGCATTCG |
| NKD-1 | GGAACTGTGCCTGCTTGACTGG | CCTGCTCACTCGCTCCTCCTC |
| Survivin | GTTAAGCAGGTGTCCAGTCCACAG | TGAATGAAGATGAGCCGCAGTGG |
| Wnt5a | CAGGTCAACAGCCGCTTCAAC | ACAATCTCCGTGCACTTCTTGC |
| DKK2 | ACAATCTCCGTGCACTTCTTGC | TGGCTTTGGAAGAGTAGGTG |
| Apc | ACAAGACGGCAGCTGGAGTATGAA | TGGATCCTGGCTATTCTTCGCTGT |
| Fzd1 | GGCCTGAAGATATGGAGTG | GGGGGAAGAAAGTAGGTTGC |
| Plcb1 | ATGAGAAGCCCAAGCTGCA | CCCTTTCATGGCTTCCTGTA |
| Wnt9a | ATG GTG TGT CTG GCT CCT G | CAG TGG CTT CAT TGG TAG TGC T |
| Rock2 | GGGATCTCATGCTGGAGTTCTTCG | TAAGGTCCTAGGTTCAATCCCCAGC |
| Lrp6 | GAGCTGGACTGTTATCCAACTG | CTTCATACGAGGACACAGCATC |
| Gsk3b | TGGAATCTGCCATCGGGATA | ATTGGGTTCTCCTCGGACCA |
